# *Gynostemma pentaphyllum* for dyslipidemia: A systematic review of randomized controlled trials

**DOI:** 10.3389/fphar.2022.917521

**Published:** 2022-08-26

**Authors:** Ning Dai, Fang-fang Zhao, Min Fang, Feng-lan Pu, Ling-yao Kong, Jian-ping Liu

**Affiliations:** ^1^ Centre for Evidence-Based Chinese Medicine, Beijing University of Chinese Medicine, Beijing, China; ^2^ Xiyuan Hospital, China Academy of Chinese Medical Sciences, Beijing, China; ^3^ Chinese Journal of Integrated Traditional and Western Medicine Press, Beijing, China; ^4^ The National Research Center in Complementary and Alternative Medicine (NAFKAM) Department of Community Medicine, Faculty of Health Science, UiT The Arctic University of Tromsø, Tromsø, Norway

**Keywords:** *Safety of Gynostemma pentaphyllum*, lipid-lowering agents, red yeast rice extracts, dyslipidemia, systematic review, meta-analysis, randomized controlled trials

## Abstract

**Objective:** To evaluate the lipid-lowering effect and safety of *Gynostemma pentaphyllum* (GP) used alone or as adjunctive therapy for dyslipidemia.

**Methods:** Eight databases and three clinical trial registries were searched until January 2022. Randomized controlled trials (RCTs) assessing the effectiveness of GP for dyslipidemia were included. Trial quality was assessed using the Cochrane Risk of Bias Tool 2.0. Data were analyzed by RevMan 5.4 with effects estimated as risk ratio (RR) or mean difference (MD) with 95% confidence intervals (CI).

**Results:** Twenty-two RCTs involving 2,407 dyslipidemia participants were included. Regarding the risk of bias, 14 RCTs had some concerns, seven RCTs were high, and one trial was low. GP was comparable to n-3 fatty acids (RR 0.89, 95% CI 0.62–1.28) and red yeast rice (RR 0.33, 95% CI 0.1–1.12) on normalization of serum lipids. GP plus n-3 fatty acid was superior in normalization of triglycerides (TG) and total cholesterol (TC) than n-3 fatty acids (RR 1.34, 95% CI 1.01–1.77). GP was similar to lipid-lowering agents (statins, fibrates, and n-3 fatty acids) in regulating TG, TC, and high-density lipoprotein cholesterol (HDL-C). GP plus lipid-lowering agents were superior to lipid-lowering agents in TG (MD −0.65 mmol/L, 95% CI −1.03 to −0.28), LDL-C (MD −0.57 mmol/L, 95% CI −1.07 to −0.08), and HDL-C (MD 0.15 mmol/L, 95% CI 0.11–0.20). GP was inferior to red yeast rice in TC (MD 0.64 mmol/L, 95% CI 0.15–1.13), TG (MD 0.43 mmol/L, 95% CI 0.15–0.71), and HDL-C (MD −0.25 mmol/L, 95% CI −0.47 to −0.04). GP had fewer adverse events than lipid-lowering drugs.

**Conclusion:** Very low certainty evidence showed that GP’s effects on TC, TG, and HDL-C were comparable to that of lipid-lowering agents. Low certainty evidence showed that red yeast rice was superior to GP in TC, TG, and HDL-C. Low to moderate certainty evidence showed that the effects of GP plus lipid-lowering agents were superior to that of lipid-lowering agents on TG, LDL-C, and HDL-C. GP use for more than 8 weeks appears safe.

**Systematic Review Registration:** https://inplasy.com/, identifier INPLASY202210135.

## Introduction

Dyslipidemia refers to an increase in the levels of total cholesterol (TC), triglyceride (TG), or low-density lipoprotein cholesterol (LDL-C) and a decrease in the level of high-density lipoprotein cholesterol (HDL-C) (Heshmat-Ghahdarijani et al., 2020). Cardiovascular diseases (CVDs) are the leading cause of morbidity and mortality globally (Chinese Medical Association, 2019; World Health Organization, 2019), while dyslipidemia is a significant risk factor for CVDs and ischemic cerebrovascular accidents (Nicholls and Lundman, 2004; Baigent et al., 2005). Dyslipidemia is one of the most critical risk factors for atherosclerosis, which can lead to cardiovascular and cerebrovascular diseases such as coronary heart disease, cerebral infarction, hypertension, and diabetes. Dyslipidemia is also a significant cause of death and disability (Anderson et al., 1987; Whelton et al., 2018). Therefore, appropriate management of dyslipidemia is essential for the primary prevention of CVDs. Epidemiological data indicated that the global prevalence of dyslipidemia in 2019 was about 15.2%, and the prevalence of dyslipidemia in adults in some developed countries was as high as 55% (Zeljković et al., 2019; Zhao, 2019; Zokaei et al., 2020). A large national cross-sectional study (*n* = 2,314,538) found that dyslipidemia is highly prevalent (33.8%) in China but commonly undertreated and uncontrolled (Lu et al., 2021). Even among people with identified arteriosclerotic cardiovascular disease (ASCVD) and a high risk of ASCVD, only 26.6 and 42.9%, respectively, achieved LDL-C control targets. Moreover, statins, the lipid-lowering agents recommended by the guidelines, are not available in nearly one-half of the primary care institutions, with the lowest available in rural village clinics. Similar data were reported in other countries, and undertreatment of dyslipidemia seems a universal problem (Lu et al., 2021). In 2019, the European Society of Cardiology (ESC) and the European Society of Atherosclerosis (EAS) published guidelines for treating dyslipidemia and suggested enhancing the management of dyslipidemia (Mach et al., 2020).

For treating dyslipidemia, dietary adjustment and physical exercise should be implemented before or at the same time as drug therapy (Whelton et al., 2018). Commonly used lipid-lowering drugs include statins, cholesterol absorption inhibitors, cholic acid chelating agent, fibrates, nicotinic acid, n-3 fatty acids, and others (Mach et al., 2020). Statins and fibrates have significant lipid-lowering effects but their adverse events include liver injury, myalgia, myositis, rhabdomyolysis, and diabetes induction (Bays et al., 2014; Maki et al., 2014; Stroes et al., 2015). Nicotinic acid has two effects. One is the vitamin potent in milligram doses and the other is the broad-spectrum lipid drug potent in gram doses, which was found in 1955 (Carlson, 2005). Two large-sample randomized controlled trials (RCTs) showed that nicotinic acid did not have any beneficial effect on lowering lipids; in contrast, they might increase the level of fasting blood glucose and glycated hemoglobin and affect the control of blood glucose in diabetic patients (Aim-High et al., 2011; Group et al., 2014). Several patients with a proven or perceived intolerance to statins and other identified lipid-lowering agents use alternative natural products to improve their lipid levels (Liu and Yu, 2016). Some natural botanical drugs, such as red yeast rice (RYR) extracts (Cicero et al., 2019) and *Hibiscus sabdariffa* (Sabzghabaee et al., 2013), have been proven beneficial for lipid modification.

*Gynostemma pentaphyllum* was initially served as a wild vegetable, as recorded in *Materia Medica for Famines* (also called ‘Jiuhuang Bencao’). The first use and therapeutic effects of *Gynostemma pentaphyllum* were recorded in a 16th-century Chinese medicine book, *Compendium of Materia Medica* (Razmovski-Naumovski et al., 2005).

*Gynostemma pentaphyllum* is geographically distributed in China, India, Nepal, Bangladesh, Sri Lanka, Myanmar, Laos, Vietnam, Malaysia, Indonesia, New Guinea, North Korea, and Japan (Ding and Zhu, 1991). It grows at 300–3,200 m above sea level in dense forests in valleys, sparse forests on hillsides, thickets, or grass on roadsides (Wang and Li, 1994; Zhao and Yan, 2020). Some commercial products from *Gynostemma pentaphyllum,* including tea and beverages, are available and beneficial for managing hyperlipidemia (Su et al., 2021). In addition, *Gynostemma pentaphyllum* was also used as additives in drinks, beer, bread, and noodles (Li et al., 2016). *Gynostemma pentaphyllum* contains more than 20 chemical components such as gynostemma saponin, especially dammarane saponins, quercetin, and sitosterol (Zheng, 2004; Nookabkaew et al., 2006; Yan et al., 2013; Jang et al., 2016). Modern pharmacological research studies provide evidence of its anti-inflammatory (Xie et al., 2010; Wong et al., 2017), anti-hyperlipidemic ability (La et al., 1995), and its regulatory role in liver function (Gou et al., 2016). In 1986, *Gynostemma pentaphyllum* was listed by the Ministry of Science and Technology as the first “precious Chinese medicine” to be developed in the “Spark Program”. Due to its extensive biological activities, *Gynostemma pentaphyllum* was brought into the list of functional foods by the Ministry of Public Health of China on 5 March 2002 (Wang et al., 2019). Currently, products containing *Gynostemma pentaphyllum* have been marketed in many Asian countries and the United States (Liu et al., 2008; Xie et al., 2012). In addition to *Gynostemma pentaphyllum*, RYR extract was approved in 1995 for the treatment of dyslipidemia, such as Xuzhikang which was recommended by clinical guidelines (Zeljko et al., 2011; Chinese Adult Dyslipidemia Guidelines Revision Joint Committee, 2016; Chinese Medical Association Branch of Integrated Traditional Chinese and Western Medicine, 2018). RYR extracts have been proven beneficial for lipid modification (Lu et al., 2008; Cicero et al., 2019). *Hibiscus sabdariffa* was also helpful for dyslipidemia (Sabzghabaee et al., 2013; Ellis et al., 2021).

However, with the wide application of lipid-lowering drugs like statins in recent years, traditional Chinese herbal lipid-lowering drugs like *Gynostemma pentaphyllum* have decreased significantly. To explore the real clinical curative effect of *Gynostemma pentaphyllum* and verify if it is still valuable as a lipid-lowering drug, we conducted this systematic review and meta-analysis of existing RCTs of *Gynostemma pentaphyllum* for the treatment of dyslipidemia.

## Objectives

This systematic review aimed to evaluate the lipid-lowering effect and safety of *Gynostemma pentaphyllum* used alone or as adjunctive therapy for the treatment of dyslipidemia in RCTs.

## Methods

### Criteria for considering studies for this review

#### Types of studies

RCTs were included irrespective of blinding, publication status, and language.

#### Types of participants

Dyslipidemia participants were included irrespective of age, gender, and the diagnostic criteria. Co-existing with other diseases (i.e.,diabetes and coronary heart diseases) were also eligible.

#### Types of interventions

All types of *Gynostemma pentaphyllum* were included, whether botanical drugs or extracts, used alone or combined with lipid-lowering agents. The duration of treatment was limited to no less than 4 weeks.

#### Types of control

The control intervention included no treatment, placebo, lipid-lowering agents including but not limited to statins, and Chinese herbal products containing RYR extracts. Western botanical drugs, which have been proved effective compared to placebo or lipid-lowering agents, were also included.

### Prespecified outcomes included

The primary outcomes sought at the end of treatment and maximal follow-up after completion of the treatment included the number of people whose lipid level returned to normal, also called normalization of lipid levels, and serum lipids, including but not limited to TC, TG, LDL-C, and HDL-C.

Secondary outcomes included major adverse cardiovascular events (fatal and non-fatal events, including myocardial infarction, angina pectoris, stroke, peripheral arterial disease, and sudden death), waist circumstances, body mass index, blood glucose, blood pressure, adverse events, and cost-effectiveness.

### Search methods for identification of studies

Eight electronic databases and three clinical trial registries, including Pubmed, Cochrane Library, Embase, Web of Science, China National Knowledge Infrastructure, Chinese Scientific Journal Database, SinoMed, Wanfang Database, World Health Organization International Clinical Trials Registry Platform (https://www.who.int/clinical-trials-registry-platform), ChilinalTrials.gov (www.clinicaltrials.gov/), and the Chinese Clinical Trial Registry (http://www.chictr.org.cn/index.aspx), were searched for published, ongoing, and unpublished trials from their inception to January 2022. The references of all identified reviews or clinical trials were searched for additional trials. Search terms, for example, ‘*Gynostemma pentaphyllum*’, ‘dyslipidemia’, and ‘randomized controlled trials’, were identified according to published systematic reviews (Ong and Aziz, 2016; Mehraban et al., 2021), clinical practice guidelines, International Classification of Diseases (ICD) -10, ICD-11., MeSH terms, and Emtree. No language restrictions were applied. The search strategies of these databases and registries were shown in Supplementary Table S1.

### Study selection and data extraction

NoteExpress (Beijing Aegean Software company, Rev 3.4.0.8878) was used to manage the electronic and manual searching results. Two authors (FFZ and FLP) independently assessed the eligibility for inclusion by screening titles, abstracts, and full text according to the prespecified selection criteria. Two authors (MF and LYK) independently extracted data with the prespecified data extraction forms, including first author, publication year, funding source, methodological characteristics, number of participants, inclusion and exclusion criteria, diagnostic criteria, intervention details, outcome measures (end of treatment and follow-up), and the number of adverse events. If the above data were not available in the trial, further information would be sought by correspondence with the principal investigator of the trials. Data from trials published in duplicate were included only once. Any disagreement was resolved by discussion or involving a third author (JPL).

### Assessment of risk of bias in included studies

Two authors (MF and LYK) independently assessed the risk of bias using the Cochrane risk-of-bias 2.0 (ROB 2) tool for each trial (Higgins et al., 2022a). The ROB 2 tool considered the following domains: bias arising from the randomization process; bias due to deviations from the intended interventions; bias due to missing outcome data; bias in the measurement of the outcome; and bias in the selection of the reported result. Any disagreement was resolved by discussion or involving a third author (JPL).

### Measures of treatment effect

Data analyses were performed by the Review Manager program (V.5.4.1 Copenhagen: The Nordic Cochrane Centre, The Cochrane Collaboration). Dichotomous data were presented as risk ratio (RR) with 95% confidence intervals (CI). Continuous data were presented as mean difference (MD) with 95% CI if data were conceptually the same but measured differently in different trials.

### Unit of analysis issues

The unit of analysis for this review was the individually randomized participants. We separated the arms into different comparisons in trials with multiple intervention groups that met the inclusion criteria. If it was not reasonable to pool the groups, we divided the ‘shared’ control group to avoid double-counting participants. In addition, if a trial reported multiple adverse events per participant, we used the total number of participants with adverse events for analysis rather than the number of adverse events.

### Dealing with missing data

We contacted investigators or authors to verify key study characteristics and obtained missing numerical outcome data when needed (e.g., when a study reported outcomes with a line chart). Whenever standard deviation was not reported by means, it was calculated from the information reported, such as CI or *p*-values (Higgins et al., 2022b). When we did not get a response, we only used the available data in the analyses.

### Assessment of heterogeneity

Heterogeneity was tested with I^2^ statistics among trials in each analysis. As recommended by the *Cochrane Handbook 6.3*, we defined I^2^ as follows: 1) less than 50%: might represent mild heterogeneity; 2) 50–70%: might represent moderate heterogeneity; 3) more than 75%: might represent severe heterogeneity (Deeks et al., 2022). A random-effects model (REM) was used when the heterogeneity was more than 75%; otherwise, a fixed-effects model (FEM) was used. We explored heterogeneity with prespecified subgroup analysis.

### Assessment of reporting biases

A Galbraith plot was used to explore publication bias. We determined publication bias statistically with a linear regression test using R 4.1.2 software.

### Assessment of quality of the evidence

The Grading of Recommendations, Assessment, Development, and Evaluation (GRADE) (Schünemann et al., 2022) is the established tool for assessing the overall certainty of evidence. GRADE is evaluated by analyzing the risk of bias, imprecision, inconsistency, indirectness, and publication bias. GRADE was used to assess the quality of included trials. This assessment was independently performed by two authors (FFZ and ND). Disagreements were resolved by discussion or involving a third author (JPL).

### Data synthesis

*Gynostemma pentaphyllum* was individually compared with each control (e.g., placebo) regardless of route of administration, dose, or preparation. We performed meta-analyses for data from similar or homogeneous trials in terms of participants, interventions, control, and outcomes. The primary analysis used a FEM. The following comparisons were calculated whenever data were available: *Gynostemma pentaphyllum* versus no intervention/placebo, *Gynostemma pentaphyllum* versus lipid-lowering agents, *Gynostemma pentaphyllum* plus lipid-lowering agents versus lipid-lowering agents, *Gynostemma pentaphyllum* versus Chinese herbal medicines which containing RYR extracts, and *Gynostemma pentaphyllum* versus *Hibiscus sabdariffa*. For dichotomous outcomes, we performed meta-analyses using the Mantel-Haenszel method. For continuous outcomes, we used the inverse-variance method.

### Subgroup analysis and investigation of heterogeneity

If sufficient data were available, the subgroup analysis would be carried out to explore any effect that might explain any heterogeneity, including the mechanism of lipid-lowering agents (fibrates versus statins), treatment duration (no more than 8 weeks versus more than 8 weeks), therapeutic method (combined with primary treatment versus without primary treatment), and comorbidities (dyslipidemia with comorbidities versus dyslipidemia without comorbidities).

### Sensitivity analysis

We performed sensitivity analyses for the primary outcome to determine whether the conclusions differed or if eligibility was restricted to trials with a low risk of overall bias. When substantial heterogeneity existed, sensitivity analysis was conducted to further investigate potential sources of heterogeneity.

### Publication bias

A Galbraith plot was used to explore publication bias. The Galbraith plot is produced by calculating the standardized estimates and dividing each estimate by its standard error (Galbraith, 1998). We assumed that the publication bias may result in an overestimation of the overall prevalence. We determined publication bias statistically by a linear regression test.

## Results

### Results of the search

**FIGURE 1:**
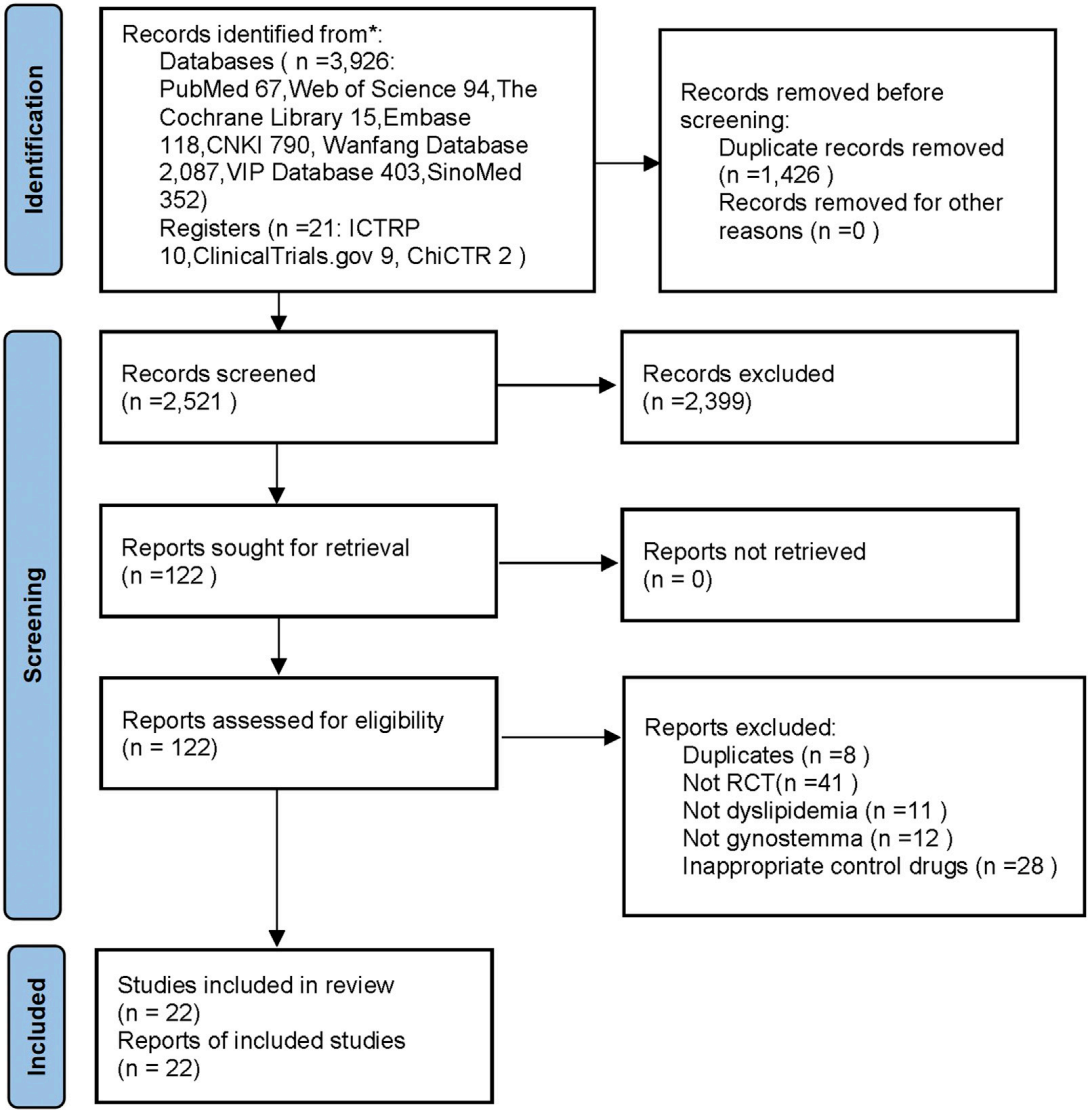
The flow diagram. Notes: CNKI: China National Knowledge Infrastructure; SinoMed: China BioMedical Literature Service System; ICTRP: the WHO International Clinical Trials Registry Platform; ChiCTR: the Chinese Clinical Trial Registry; n: number; RCT: randomized controlled trails.

From eight databases and three clinical trial registries, 3,947 records were obtained. After moving 1,426 duplicates, 2,399 records were excluded after screening titles and abstracts. Then, 122 records remained and were downloaded in full text and screened. Finally, 22 trials met the inclusion criteria and were used for meta-analyses. The flow diagram was shown in Figure 1.

### Description of included trials

Table 1 showed the characteristics of the 22 trials published from 1996 to 2016, including 2,407 participants aged 19 to 71. Fourteen trials reported that participants with dyslipidemia had comorbidities, such as hypertension, coronary heart disease, and diabetes (Lu et al., 1996; Chen et al., 1998; Fu et al., 2000; Zhang et al., 2000; Zhang and Tang, 2000; Li et al., 2001; Lin, 2001; Zhao et al., 2009; Peng, 2010; Wang and Du, 2010; Chen, 2011; Xing et al., 2013; Shi, 2016; Shi and Fang, 2016). Dyslipidemia participants in one trial had no other comorbidities (Yu et al., 1997), and seven trials did not report comorbidities. Two trials were published in English (Wang et al., 1997; Jeenduang et al., 2017) and 20 in Chinese. Three trials compared *Gynostemma pentaphyllum* plus lipid-lowering drugs with lipid-lowering drugs (Lu, 2005; Xu, 2013; Shi and Fang, 2016), and seven trials compared *Gynostemma pentaphyllum* with lipid-lowering drugs (Chen et al., 1998; Li et al., 2001; Zhou, 2005; Huang, 2006; Zhao et al., 2009; Peng, 2010; Shi, 2016). Eight trials compared *Gynostemma pentaphyllum* with RYR extracts (Lu et al., 1996; Liu and Zhang, 1997; Wang et al., 1997; Yu et al., 1997; Fu et al., 2000; Zhang and Tang, 2000; Wang and Du, 2010; Chen, 2011), and one trial compared *Gynostemma pentaphyllum* with *Hibiscus sabdariffa* (Nicholls and Lundman, 2004). There were two three-arm RCTs (Zhang et al., 2000; Lin, 2001) and one four-arm RCT (Xing et al., 2013) in the review. *Gynostemma pentaphyllum* in 21 RCTs came from China (eight from Shanxi province, two from Guangdong province, one from Jiangxi province, one from Zhejiang province, and nine trials did not report the province of origin), and one came from Thailand. *Gynostemma pentaphyllum* from China and Thailand belonged to the same species. The botanical drug preparations reported in the included trials were shown in Supplementary Table 2.

**TABLE 1:** Characteristics of the included 22 studies.

Study ID	Setting	Funding sources	Age: Mean ± SD (y)		Sample size (M/F)		Comorbidities	*Gynostemma pentaphyllum* intervention	Details of the control group	Basic treatment	Treatment duration	Outcome measures
			T	C	T	C						
Chen (2011)	Outpatient	NR	median51 (range35–67)	median52 (range35–69)	31/17	31/21	Hypertension, coronary heart disease, and diabetes	Gypenosides tablet (3 tablets) po tid	Red yeast rice (Zhibituo) 1.05g, po, tid	Complications were routinely treated	2 m	TC, TG, LDL-C, HDL-C, effective rate, AE
Chen et al. (1998)	Inpatient	NR	54 (40–70)	54 (40–70)	18	22	Diabetes and fatty liver	Gypenosides tablet 40 mg po tid	Lovastatin 20 mg po qd	Control blood glucose	8w	TC, TG, LDL-C, HDL-C, blood glucose, AE
Fu et al. (2000)	NR	NR	NR	mean59.8 (range43–78)	160		Hypertension, coronary heart disease, and diabetes	Gypenosides 60 mg po tid	Red yeast rice (Zhibituo) 1.05 g, po, tid	NR	2–12 m	TC, TG, HDL-C
Huang (2006)	NR	NR	60.2 ± 9.4 (45–75)	59 ± 8.7 (48–73)	18/12	20/10	NR	Gypenosides tablet 60 mg po tid	Simvastatin tablet 20 mg po tid	NR	12w	TC, TG, LDL-C, HDL-C, AE
Jeenduang et al. (2017)	NR	University	NR	NR	17	31	NR	*Gynostemma pentaphyllum* tea 3 g was added to 240 ml of boiling water, po, bid	*Hibiscus* sabdariffa tea 3 g was added to 240 ml of boiling water, po, bid	NR	30 d	TC, TG, LDL-C, HDL-C, waist circumstances, body mass index**,** blood glucose, BP, AE
Li et al. (2001)	Inpatient	NR	55.2 (40–76)	51.3 (34–73)	20/14	21/9	Gastric cancer, colorectal cancer, primary liver cancer, lung cancer, breast cancer, hypertension, coronary heart disease, diabetes, cholecystitis, cholelithiasis, and fatty liver	Gypenosides capsules 40mg, po, tid	N-3 fatty acids 0.9g, po,tid	NR	2 m	AE
Lin (2001)	Outpatient + Inpatient	NR	45 ± 9	45 ± 9	19	26	Diabetes, severe liver and kidney dysfunction, and endocrine disorders	Gypenosides tablet 80 mg, po, bid; Gypenosides tablet 80 mg, po, qd + Fenofibrate 200 mg, po, bid	Fenofibrate 200 mg, po, qd	NR	12w	TC, TG, LDL-C, HDL-C, AE
Liu and Zhang, (1997)	NR	NR	40–81	42–82	16/14	19/11	NR	Gypenosides tablet (40 mg) po, tid	Red yeast rice (Zhibituo) 1.05 g, po, tid	NR	4w	normalization of lipid levels
Lu (2005)	Outpatient	NR	28–76	30–75	26/24	25/25	NR	*Gynostemma pentaphyllum* tea 30 g in boiling water to drink + N-3 fatty acids 0.5g, po,tid	N-3 fatty acids, 0.5 g, po, tid	NR	3 m	Effective rate
Lu et al. (1996)	Outpatient + Inpatiet	NR	mean56.9 (range 37–70)	mean56.9 (range 37–70)	63	58	Hypertension and coronary heart disease	Gypenosides tablet 60 mg po bid	Red yeast rice (Xuezhikang capsule) 0.6 g po bid	Continue to take drugs that do not affect lipid metabolism	8w	TC, TG, LDL-C, HDL-C, Effective rate, AE
Peng (2010)	NR	NR	52.8 (44–67)	54.2 (42–68)	27/15	28/16	Hypertension, coronary heart disease, diabetes, fat liver, and hypothyroidism	Gypenosides 40 mg, po tid	Fenofibrate 10 mg, po, tid	Control diet, increase physical activity, control blood pressure and blood glucose	4w	TC, TG, LDL-C, HDL-C, AE
Shi (2016)	Outpatient	Government	52.72 ± 9.41	54.94 ± 8.67	24/38	22/41	Hypertension, coronary heart disease, diabetes, and stroke	Gypenosides 60 mg po tid	Simvastatin capsule 20 mg, po, qn	NR	12w	TC, TG, LDL-C, HDL-C, Effective rate, AE
Shi and Fang (2016)	NR	NR	52.05 ± 7.93	51.45 ± 8.12	178		Diabetes	Gynostemma pentaphyllum powder 6 g + Metformin 0.5 g + Atorvastatin 20mg, po tid	Metformin 0.5 g + Atorvastatin 20 mg, po tid	Basic diabetes education, diabetes diet, moderate exercise, and other lifestyle intervention for patients with diabetes	8w	TC, TG, LDL-C, HDL-C
Wang et al. (1997)	NR	University	56.4 ± 0.83	56.0 ± 0.50	73/49	188/136	NR	Gypenosides tablet (0.6 g), po, bid	Red yeast rice 0.6 g, po, bid	All medications were allowed during the trial, except those that could affect serum lipids	8w	normalization of lipid levels, TC, TG, LDL-C, HDL-C, Effective rate, AE
Wang and Du, (2010)	Outpatient + Inpatiet	NR	54.9 ± 3.21 (25.7–75.4)	43.7 ± 3.15 (22.5–76.2)	23/10	30/42	Hypertension and coronary heart disease	Gypenosides tablet 120 mg po tid	Red yeast rice (Xuezhikang capsule) 0.6 g po bid	Active treatment of the primary disease (hypertensive disease and coronary heart disease)	8w	TC, TG, LDL-C, HDL-C, Effective rate, AE
Xing et al. (2013)	Outpatient + Inpatient	NR	60.0 ± 10.2 61.0 ± 9.9 62.0 ± 9.1	63.0 ± 8.9	44/46	15/15	Hypertension, diabetes, stroke, and heart failure	Gypenosides table 60mg, po, tid Gypenosides table 120 mg, po, tid; Gypenosides table 120 mg po tid + Atorvastatin 20 mg po qd	Atorvastatin 20 mg po qd	NR	1 m	TC, TG, LDL-C, HDL-C, AE
Xu (2013)	Outpatient	NR	NR	NR	50	46	NR	Gypenosides tablet 20 mg + Simvastatin capsule 20 mg, po, tid	Simvastatin capsule 20 mg, po, tid	NR	8w	TC, TG, LDL-C, HDL-C, Effective rate
Yu et al. (1997)	Outpatient	NR	52.6 ± 10.4 (27–67)	53.9 ± 9.6 (32–70)	20/10	57/31	No.	Gypenosides 60 mg po bid	Red yeast rice (Xuezhikang capsule) 0.6 g po bid	Low fat, low cholesterol diet, lifestyle remained relatively stable	8w	TC, TG, LDL-C, HDL-C, Apo A1, Apo B, Effective rate, AE
Zhang and Tang (2000)	NR	NR	56 ± 9 (40–74)	59 ± 6 (42–73)	19/34	24/19	Hypertension, coronary heart disease, and stroke	Gypenosides tablet 60 mg po tid	Red yeast rice (Xuezhikang capsule) 0.6 g po bid	NR	4w	TC, TG, LDL-C, HDL-C, AE
Zhang et al. (2000)	Outpatient	NR	60–79	60–79	140	6	Hypertension, coronary heart disease, diabetes, fat liver, and cerebral infarction	Gypenosides tablet (60 mg) po, tid; Gypenosides tablet (40 mg) + Gypenosides gelatin pearl (1.35 g) + N-3 fatty acids, capsule 1.8g, po, tid	N-3 fatty acids, capsule 1.8 g, po, tid	NR	60 d	normalization of lipid levels
Zhao et al. (2009)	Outpatient + Inpatient	NR	41.23 ± 8.34 (19–64)	40.13 ± 8.45 (18–65)	17/13	18/12	Idiopathic nephrotic syndrome	Gypenosides capsule 60 mg po tid	Simvastatin tablet 20 mg po tid	Active treatment of the primary disease (hormones, anticoagulants, etc.), in addition, eating habits and lifestyle remain the same as before treatment	4w	TC, TG, LDL-C, HDL-C, AE
Zhou (2005)	NR	NR	52 ± 7.7 (35–70)	52 ± 7.7 (35–70)	53	29	NR	Gypenosides tablet 120 mg po tid	Gemfibrozil capsule 0.6 g po bid	NR	3 m	Effective rate, AE

Note: T: treatment group; C: control group; M: male; F: female; y: years; m: months; w: weeks; d: days; NR: Not reported. Po: peros; qd: once a day; tid: three times a day; bid: twice a day; mg: milligram; g: Gram; TC: total cholesterol; TG: triglycerides; LDL-C: low-density lipoprotein cholesterol; HDL-C: high-density lipoprotein cholesterol; AE: adverse events; BP: blood pressure.

Three trials reported normalization of lipid levels (Liu and Zhang, 1997; Wang et al., 1997; Zhang et al., 2000), and most trials reported TG level, TC level, LDL-C level, HDL-C level, and adverse events. No trial reported significant adverse cardiovascular events or cost-effectiveness. Two trials were supported by universities (Wang et al., 1997; Jeenduang et al., 2017), one was supported by the government (Shi, 2016), and the remaining trials did not report support.

### Risk of bias in included studies

Three trials reported the normalization of lipids (Liu and Zhang, 1997; Wang et al., 1997; Zhang et al., 2000). As for the overall risk of bias, one trial was assessed as low risk of bias; another trial was considered as a medium risk because it did not report the random sequence generation method, and a third trial was assessed as high risk of bias because of the wrong random and selection of the reported result.

Eighteen trials reported the serum lipids (Lu et al., 1996; Wang et al., 1997; Yu et al., 1997; Chen et al., 1998; Fu et al., 2000; Zhang and Tang, 2000; Li et al., 2001; Lin, 2001; Zhou, 2005; Huang, 2006; Zhao et al., 2009; Peng, 2010; Wang and Du, 2010; Chen, 2011; Xing et al., 2013; Xu, 2013; Shi, 2016; Shi and Fang, 2016; Jeenduang et al., 2017). Regarding overall bias, 13 trials had some concerns, and five were assessed as having a high risk of bias. As for the randomization process, all trials were described as “randomized” but did not report a blinding method. So, they had some concerns. Regarding bias due to deviations from intended interventions, four trials were assessed as high risk of bias because they did not use appropriate analysis methods to estimate the effect of assignment to intervention, and there were missing data (Lu et al., 1996; Wang et al., 1997). Two trials had some concerns because they did not use appropriate analysis methods to estimate the effect of assignment to intervention (Shi, 2016; Jeenduang et al., 2017). The remaining trials were assessed as having a low risk of bias. All trials were considered to have a low risk of bias in the domain of missing outcome data and the domain of bias in the outcome measurement. One trial did not report all results measured at all times, so it was assessed as having a high risk of bias in selecting the reported result (Shi, 2016). However, 18 trials were considered to have a low risk of bias in this domain (see Table 2 and Supplementary Figure S1).

**TABLE 2:** Risk of bias of the 22 included randomized trials on *Gynostemea pentaphyllum* for dyslipidemia.

Domains	Low risk of bias n (%)	Some concerns n (%)	High risk of bias n (%)
Bias arising from the randomization process	1 (4.5)	20 (91)	1 (4.5)
Bias due to deviations from the intended interventions	16 (72.7)	2 (9.1)	4 (18.2)
Bias due to missing outcome data	22 (100)	0 (0)	0 (0)
Bias in the measurement of the outcome	21 (95.5)	0 (0)	1 (4.5)
Bias in the selection of the reported result	19 (86.4)	0 (0)	3 (13.6)
The overall risk of bias	1 (4.5)	14 (63.6)	7 (31.8)

One trial reported a cure rate (Lu, 2005). It had some concerns in the randomization process domain because it did not report the random sequence generation method. Therefore, it was assessed as a high risk of bias because of the selection of the reported result (see Table 2).

### Effects of interventions

#### *Gynostemma pentaphyllum* versus lipid-lowering agents (11 Trials)

Compared with n-3 fatty acids, *Gynostemma pentaphyllum* showed no significant difference in the normalization of lipid levels (RR 0.89, 95% CI 0.62 to 1.28; 1 trial, 96 participants) (Zhang et al., 2000).

There was no significant difference between the *Gynostemma pentaphyllum* group and the lipid-lowering agents (statins, fibrates, and n-3 fatty acids) in TG, TC, or HDL-C levels. However, *Gynostemma pentaphyllum* showed a less beneficial effect on LDL-C level than statins and fenofibrate (Table 3).

**TABLE 3:** The effects of *Gynostemma Pentaphyllum* on dyslipidemia from 22 randomized controlled trials.

Outcome	Control	No. of studies	No. of participants	I^2^, model (REM/FEM)	Effect size MD mmol/L [95% CI]	Quality of evidence (GRADE)
***Gynostemma Pentaphyllum* versus control**						
TC level	Lipid-lowering agents	7	491	90%, REM	0.52 [−0.01, 1.04]	Very low certainty
	Red yeast rice	5	859	96%, REM	0.64 [0.15, 1.13]	Low certainty
	*Hibiscus sabdariffa*	1	48	0%, FEM	−0.42 [−0.97, 0.13]	Low certainty
TG level	Lipid-lowering agents	7	491	91%, REM	0.13 [−0.21,0.47]	Very low certainty
	Red yeast rice	5	859	96%, REM	0.43 [0.15, 0.17]	Low certainty
	*Hibiscus sabdariffa*	1	48	0%, FEM	−0.24 [−0.62, 0.15]	Low certainty
LDL-C level	Lipid-lowering agents	6	461	84%, REM	0.57 [0.20, 0.93]	Low certainty
	Red yeast rice	3	621	99%, REM	0.37 [−0.46, 1.20]	Very low certainty
	*Hibiscus sabdariffa*	1	48	0%, FEM	−0.32 [−0.77, 0.13]	Low certainty
HDL-C level	Lipid-lowering agents	7	491	99%, REM	−0.34 [−0.93, 0.25]	Very low certainty
	Red yeast rice	4	781	96%, REM	−0.25 [−0.47, −0.04]	Low certainty
	*Hibiscus sabdariffa*	1	48	50%, FEM	0.10 [−0.08, 0.28]	Low certainty
***Gynostemma pentaphyllum* plus lipid-lowering agents versus lipid-lowering agents**						
TC level	Lipid-lowering agents	4	364	98%, REM	−1.05 [−2.28, 0.19]	Very low certainty
TG level	Lipid-lowering agents	4	364	92%, REM	−0.65 [−1.03, −0.28]	Low certainty
LDL-C level	Lipid-lowering agents	3	334	85%, REM	−0.57 [−1.07, −0.08]	Moderate certainty
HDL-C level	Lipid-lowering agents	4	364	71%, REM	0.15 [0.11, 0.20]	Low certainty

Note: GP: *Gynostemma pentaphyllum;* No: number; REM: random-effects model; FEM: fixed-effects model; MD: mean difference; CI: confidence interval; TC:total cholesterol; TG:triglyceride; LDL-C: low-density lipoprotein cholesterol; HDL-C: high-density lipoprotein cholesterol.

*Gynostemma pentaphyllum* was more effective than lovastatin in reducing blood glucose level in patients with dyslipidemia combined with diabetes (MD −2.10 mmol/L, 95% CI −3.13 to −1.07; 1 trial, 40 participants) (Chen et al., 1998).

#### *Gynostemma pentaphyllum* versus red yeast rice extracts (8 Trials)

There was no significant difference between RYR and *Gynostemma pentaphyllum* in the normalization of lipid level (RR 0.33, 95% CI 0.10 to 1.12; 2 trials, 506 participants) (Liu and Zhang, 1997; Wang et al., 1997).

There was no significant difference between *Gynostemma pentaphyllum* and RYR in LDL-C level. *Gynostemma pentaphyllum* showed a negative effect compared to RYR on TC, TG, and HDL-C levels (Table 3). A subgroup analysis of treatment duration showed that *Gynostemma pentaphyllum* might be inferior to RYR in TC with more than 8 weeks of treatment. There was no significant difference between *Gynostemma pentaphyllum* and RYR in TC within 8 weeks of treatment. A subgroup analysis according to comorbidities showed that *Gynostemma pentaphyllum* might be inferior to RYR in TG of both dyslipidemia participants without comorbidities and dyslipidemia participants with comorbidities (see Supplementary Table 3).

#### *Gynostemma pentaphyllum* versus *hibiscus sabdariffa* (1 trial)

One trial compared *Gynostemma pentaphyllum* with *Hibiscus sabdariffa* and reported results according to genotype. There was no significant difference between *Hibiscus sabdariffa* and *Gynostemma pentaphyllum* in the TC level, TG level, LDL-C level, HDL-C level, waist circumstances, body mass index, or blood pressure regardless of the presence of Apolipoprotein E (APOE) genotype or *CETP TaqIB* genotype (Jeenduang et al., 2017) (Table 3). However, *Gynostemma pentaphyllum* showed an inferior effect on blood glucose compared with *Hibiscus sabdariffa* according to *CETP TaqIB* genotype (MD 0.27 mmol/L, 95% CI 0.04 to 0.50; 1 trial, 48 participants).

#### *Gynostemma pentaphyllum* plus lipid-lowering agents versus lipid-lowering agents (5 Trials)

Compared with n-3 fatty acids, *Gynostemma pentaphyllum* plus n-3 fatty acids showed a beneficial effect on the normalization of TC and TG levels (RR 1.34, 95% CI 1.01 to 1.77; 1 trial, 98 participants) (Zhang et al., 2000).

There was no significant difference between the *Gynostemma pentaphyllum* plus lipid-lowering agents group and the lipid-lowering agents' group at the TC level. Compared with lipid-lowering agents, *Gynostemma pentaphyllum* plus lipid-lowering agents showed beneficial effects on TG, LDL-C, and HDL-C levels (Table 3). A subgroup analysis of treatment duration showed that *Gynostemma pentaphyllum* plus statins might be superior to statins in the TG level within no more than 8 weeks of treatment. *Gynostemma pentaphyllum* plus fenofibrate might be superior to fenofibrate in TG level with more than 8 weeks of treatment. A subgroup analysis according to comorbidities showed that simvastatin plus *Gynostemma pentaphyllum* might be superior to simvastatin in LDL-C level of dyslipidemia participants without comorbidities. However, there was no significant difference between atorvastatin plus *Gynostemma pentaphyllum* and atorvastatin in the LDL-C level of dyslipidemia participants with comorbidities (see Table 2).

### Safety of *Gynostemma pentaphyllum*

Fifteen RCTs reported adverse events. There were no trials that reported serious adverse events. Non-serious adverse events included symptoms and abnormal laboratory indicators. The symptoms included abdominal distention, abdominal pain, headache, muscle pain, upper abdomen distention, stomach distention, dry stool, dizziness, nausea, rash, diarrhea, and epigastric burn. The abnormal laboratory indicators included abnormal liver function and abnormal creatine kinase. The adverse event rate of *Gynostemma pentaphyllum* was significantly lower than that of lipid-lowering agents (incidence of adverse event 6.5% versus 21.2%; RR 0.35, 95% CI 0.20 to 0.60; 9 trials, 607 participants), including statins, fibrates, and n-3 fatty acids. However, *Gynostemma pentaphyllum* plus lipid-lowering agents showed no additional increase of adverse events compared with lipid-lowering agents (incidence of adverse event 8.1% versus 21.1%; RR 0.44, 95% CI 0.15 to 0.35; 2 trials, 90 participants). *Gynostemma pentaphyllum* also showed no additional increase of adverse events compared with RYR preparation (Xuezhikang and Zhibituo) (incidence of adverse event 6% versus 4.0%; RR 0.52, 95% CI 0.24 to 1.12; 46 trials, 937 participants).

We planned to extract information about drug−drug interaction. However, none of the included trials reported the information on herb−drug interactions in the Methods and the Results sections.

### Publication bias

**FIGURE 2:**
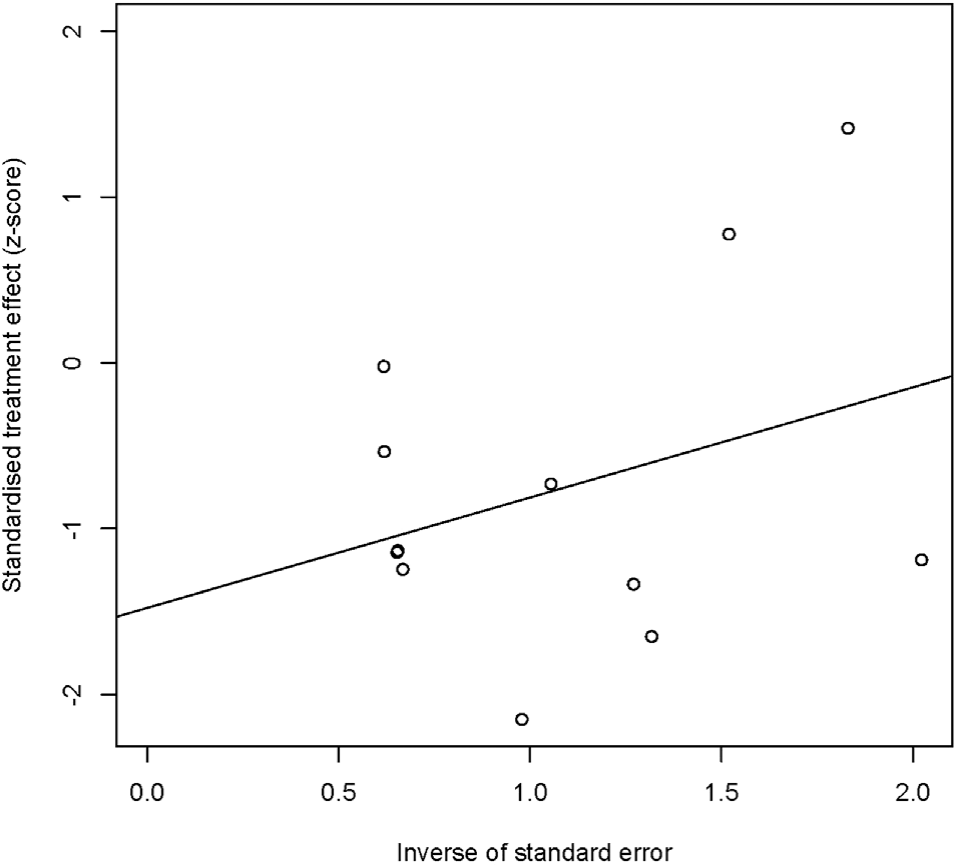
Publication bias of the included trials.

A Galbraith plot (Figure 2) demonstrated the symmetry of studies for dyslipidemia. Publication bias was not found after using the linear regression test (Egger’s method). The bias (intercept) estimate amounted to -2.00 with a standard error of 10 (*p*-value = 0.0733).

### Certainty of evidence

Very low certainty evidence showed that *Gynostemma pentaphyllum*’s effects on TC, TG, and HDL-C levels were comparable to that of lipid-lowering agents. Very low certainty evidence showed that *Gynostemma pentaphyllum*’s effects on lipid normalization and the LDL-C level were similar to that of RYR preparation. Low certainty evidence showed that RYR was superior to *Gynostemma pentaphyllum* in TC, TG, and HDL-C levels. Very low certainty evidence showed that the effect of *Gynostemma pentaphyllum* plus lipid-lowering agents were comparable to lipid-lowering agents on the TC level. From low to moderate certainty, evidence showed that the effects of *Gynostemma pentaphyllum* plus lipid-lowering agents were superior to that of lipid-lowering agents on TG, LDL-C, and HDL-C levels (Table 3).

## Discussion

### Summary of main results

Twenty-two RCTs involving 2,407 dyslipidemia participants were included in this review of *Gynostemma pentaphyllum*. The included RCTs were conducted in China (*n* = 21) or Thailand (*n* = 1), and published in English (*n* = 2) and Chinese (*n* = 20). The median treatment duration of these 22 RCTs was 8 weeks (4–48 weeks). There was no placebo-controlled trial identified in this review. Ten RCTs compared *Gynostemma pentaphyllum* with lipid-lowering agents recommended by the guidelines, including lovastatin, simvastatin, n-3 fatty acid, fenofibrate, and gemfibrozil. Six RCTs compared *Gynostemma pentaphyllum* plus lipid-lowering agents with lipid-lowering agents, including fenofibrate, n-3 fatty acid, atorvastatin, and simvastatin. Seven RCTs compared *Gynostemma pentaphyllum* with RYR preparation. This review identified three *Gynostemma pentaphyllum* preparations (*Gynostemma pentaphyllum* tea, gypenosides capsules and tablets). A meta-analysis of 10 RCTs showed that *Gynostemma pentaphyllum* was less effective than lipid-lowering agents on the LDL-C level. Compared with n-3 fatty acids, *Gynostemma pentaphyllum* plus n-3 fatty acids showed a beneficial effect on the normalization of TC and TG levels. Low to moderate evidence showed that *Gynostemma pentaphyllum* plus lipid-lowering agents had beneficial effects on TG, LDL-C, and HDL-C levels compared with lipidlowering agents, including fenofibrate, atorvastatin, and simvastatin. Low evidence showed that *Gynostemma pentaphyllum* was less effective than RYR in TC, TG, and HDL-C levels.

Regarding safety, *Gynostemma pentaphyllum* had fewer adverse events than lipid-lowering drugs. No trial reported a serious adverse event. Non-serious adverse events reported in the 15 RCTs included abdominal pain, headache, occasional liver dysfunction, and abnormal creatine kinase. Compared with lipid-lowering agents (statins and fibrates), the combination of *Gynostemea pentaphyllum* with lipid-lowering agents for 8 weeks did not increase the additional benefit of lipid-lowering agents. Compared with RYR, *Gynostemea pentaphyllum* also did not increase any other benefit.

### Quality of the evidence

We judged the overall certainty of evidence to be very low for the TC, TG, LDL-C, and HDL levels when *Gynostemma pentaphyllum* was compared with lipid-lowering agents.

### Strengths and limitations

This might be the first systematic review to assess the effect of *Gynostemma pentaphyllum* on dyslipidemia. We followed the Cochrane methodology and registered online for the protocol for the systematic review. We extensively searched using different databases to look for RCTs without language restrictions. Since all the authors responsible for the search were Chinese, there might be a bias in studies published in Chinese or Chinese journals as they seem more accessible for analysis than other articles in non-English languages. However, there were no applied language restrictions. There might be some limitations because publication bias could not be excluded entirely, and the results should be interpreted with caution.

### Comparison with other studies or reviews

Previous studies have shown that *Gynostemma pentaphyllum* extracts were more beneficial than placebo in reducing TC, TG, and LDL-C levels of overweight or obese participants (Park et al., 2013; Rao et al., 2021). This review included participants with dyslipidemia who failed to find statistically significant effects of *Gynostemma pentaphyllum* and lipid-lowering agents on serum lipids such as TC, TG, and HDL-C levels. However, *Gynostemma pentaphyllum* plus lipid-lowering agents showed more beneficial effects on TG, LDL-C, and HDL-C levels than lipid-lowering agents.

Previous studies showed that combining *Gynostemma pentaphyllum* and simvastatin could lower lipid levels by suppressing the increased expression of PCSK9 and reducing the degradation of low-density lipoprotein receptors. After combining *Gynostemma pentaphyllum* and simvastatin, the increase in serum transaminase induced by simvastatin was reversed, and liver function was improved (Wu and Qian, 2017; Su et al., 2021; Wang et al., 2021). The potential mechanism of lowering transaminase from *Gynostemma pentaphyllum* is probably due to liver function protection (Bae et al., 2018; Shen et al., 2020).

A previous systematic review found no difference in TC, TG, LDL-C, or HDL-C levels between RYR and statins (Ong and Aziz, 2016). This review found no difference in TG, TC, or HDL-C levels between *Gynostemma pentaphyllum* and lipid-lowering agents. There was also no difference in LDL-C levels between *Gynostemma pentaphyllum* and RYR. However, RYR seemed superior to *Gynostemma pentaphyllum* in regulating TC, TG, and HDL-C levels.

Although statins are effective in lowering lipid, they also have some side effects, the most common of which is liver dysfunction (1.9–5.5%) (Björnsson, 2017; Chinese Medical Association, 2019). In this review, the common adverse events of *Gynostemea pentaphyllum* included abdominal pain and abdominal distention. The adverse event rate of *Gynostemma pentaphyllum* was significantly lower than that of lipid-lowering agents. It might be because *Gynostemea pentaphyllum* was a botanical drug. The chemical constituents in *Gynostemma pentaphyllum* included gypenosides, polysaccharides, flavonoids, phytosterols, amino acids, and inorganic elements, which have the function of regulating blood lipids, resisting atherosclerosis, protecting the liver, and lowering blood sugar (Su et al., 2021). No serious adverse effects from *Gynostemea pentaphyllum* were documented in the included trials.

Current studies concluded that gypenosides were the main effective components of *Gynostemma pentaphyllum* to lower lipids (Lee et al., 2019), while gypenosides are thermally unstable substances (Wu et al., 2014). Some researchers have pointed out that high temperatures could destroy the functional components in *Gynostemma pentaphyllum*; the water temperature for *Gynostemma pentaphyllum* tea should be 60–70°C (Li and Zhang, 2018).

This review found that *Gynostemma pentaphyllum* tea with boiling water showed better lipid-lowering effects than *Hibiscus sabdariffa*. Therefore, it is speculated that in addition to gypenosides, there are other effective lipid-lowering ingredients in *Gynostemma pentaphyllum*.

### Implications for future studies

Compared with lipid-lowering agents, *Gynostemma pentaphyllum* used alone or as an adjunctive therapy could regulate the TC level, TG level, or HDL-C level of dyslipidemia participants for more than 8 weeks. The main goal of treating dyslipidemia is to avoid cardiovascular disease events, which unfortunately were not observed in all included studies. Future clinical studies on the effectiveness of natural pharmaceuticals need to consider cardiovascular events as outcome indicators. For dyslipidemia participants complicated with diabetes or other diseases, outcomes such as blood glucose and body weight should also be paid attention to. Future well-designed trials, especially randomized placebo-controlled trials, are needed to evaluate the effectiveness and safety of *Gynostemma pentaphyllum* for dyslipidemia and to report according to the CONSORT checklist (Schulz et al., 2010).

### Implications for practice

The commonly used dosage forms of *Gynostemma pentaphylla* include tea, powder, capsule, and tablet. *Gynostemma pentaphyllum* or *Gynostemma pentaphyllum* plus lipid-lowering agents should be used to regulate the TC level, TG level, or HDL-C level of dyslipidemia participants for more than 8 weeks. Compared with lipid-lowering agents, *Gynostemma pentaphyllum* has similar effects on TC, TG, and HDL-C levels and has fewer adverse events. *Gynostemma pentaphyllum* was superior to lovastatin in lowering blood glucose in diabetes patients complicated with dyslipidemia. Compared with botanical drugs such as RYR preparation, *Gynostemma pentaphyllum* showed similar effects on the LDL-C level and adverse events. However, RYR preparation showed superior to *Gynostemma pentaphyllum* in TC, TG, and HDL-C levels. Compared with lipid-lowering agents, such as statins and fibrates, *Gynostemma pentaphyllum* was superior in lowering TG and LDL-C levels and increasing HDL-C levels. *Gynostemma pentaphyllum* can be used as an alternative treatment for dyslipidemia patients to regulate TC, TG, and HDL-C levels.

## Conclusion

Very low certainty evidence showed that *Gynostemma pentaphyllum*’s effects on TC, TG, and HDL-C levels were comparable to that of lipid-lowering agents. Low certainty evidence showed that RYR was superior to *Gynostemma pentaphyllum* in TC, TG, and HDL-C levels. Low to moderate certainty evidence showed that the effects of *Gynostemma pentaphyllum*’s plus lipid-lowering agents were superior to that of lipid-lowering agents on TG, LDL-C, and HDL-C levels. Its use for more than 8 weeks appears to be safe.

## Data Availability

The original contributions presented in the study are included in the article/Supplementary Material, further inquiries can be directed to the corresponding author.

## Abbreviations

TC, total cholesterol; TG, triglyceride; LDL-C, low-density lipoprotein cholesterol; HDL-C, high-density lipoprotein cholesterol; CVDs, cardiovascular diseases; ASCVD, arteriosclerotic cardiovascular disease; ESC, the European Society of Cardiology; EAS, the European Society of Atherosclerosis; RCTs, randomized controlled trials; RYR, red yeast rice; ICD, International Classification of diseases; ROB 2, the Cochrane risk-of-bias 2.0; RR, risk ratio; CI, confidence intervals; MD, mean difference; SMD, standardized mean difference; GRADE, the Grading of Recommendations, Assessment, Development, and Evaluation.
