# Supplementary material for: Gynostemma pentaphyllum for dyslipidemia: A systematic review of randomized controlled trials
Source: Front Pharmacol. 2022 Aug 26;13:917521. doi: 10.3389/fphar.2022.917521 (PMC9459123; doi:10.3389/fphar.2022.917521)
Supplement: Supplementary file 1 [file Table2.DOCX]

**Table 1 The characteristics of the included 22 studies**

| **Study ID** | **Setting** | **Funding sources** | **Age: Mean±SD (y)** | | **Sample size (M/F)** | | **Comorbidities** | **Gynostemma pentaphyllum intervention** | **Details of control group** | **Basic treatment** | **Treatment duration** | **Outcome measures** |
| --- | --- | --- | --- | --- | --- | --- | --- | --- | --- | --- | --- | --- |
|  |  |  | T | C | T | C |  |  |  |  |  |  |
| Chen HJ 2001 | Outpatient | NR | median51(range35-67) | median52(range35-69) | 31/17 | 31/21 | Hypertension, coronary heart disease, and diabetes. | Gypenosides tablet (3 tablets) po tid | Red yeast rice (Zhibituo) 1.05g, po, tid. | Complications were routinely treated | 2m | TC, TG, LDL-C, HDL-C, Effective rate, AE |
| Chen HW 1998 | Inpatient | NR | 54(40-70) | 54(40-70) | 18 | 22 | Diabetes and fat liver. | Gypenosides tablet 40mg po tid | Lovatatin 20mg po qd | Control blood glucose. | 8w | TC, TG, LDL-C, HDL-C, blood glucose, AE |
| Fu GX 2000 | NR | NR | NR | mean59.8(range43-78) | 160 | | Hypertension, coronary heart disease, and diabetes. | Gypenosides 60mg po tid | Red yeast rice (Zhibituo) 1.05g, po, tid. | NR | 2-12m | TC, TG, HDL-C |
| Huang XP 2006 | NR | NR | 60.2±9.4(45-75) | 59±8.7(48-73) | 18/12 | 20/10 | NR | Gypenosides tablet 60mg po tid | Simvastatin tablet 20mg po tid | NR | 12w | TC, TG, LDL-C, HDL-C, AE |
| Jeenduang N 2017 | NR | University | NR | NR | 17 | 31 | NR | Gynostemma pentaphyllum tea 3g was added to 240 mL of boiling water, po, bid. | *Hibiscus sabdariffa* tea 3g was added to 240 mL of boiling water, po, bid. | NR | 30d | TC, TG, LDL-C, HDL-C, waist circumstances, body mass index, blood glucose, BP, AE |
| Li HC 2001 | Inpatient | NR | 55.2(40-76) | 51.3(34-73) | 20/14 | 21/9 | Gastric cancer, colorectal cancer, primary liver cancer, lung cancer, breast cancer, hypertension, coronary heart disease, diabetes, cholecystitis cholelithiasis, and fatty liver. | Gypenosides capsules 40mg, po, tid | N-3 fatty acids~~Doxicon~~ 0.9g, po ,tid. | NR | 2m | AE |
| Lin ZD 2001 | Outpatient+Inpatiet | NR | 45±9 | 45±9 | 19 | 26 | Diabetes, severe liver and kidney dysfunction, and endocrine disorders | Gypenosides tablet 80mg, po, bid; Gypenosides tablet 80mg, po, qd + Fenofibrate 200mg, po, bid. | Fenofibrate 200mg, po, qd. | NR | 12w | TC, TG, LDL-C, HDL-C, AE |
| Liu CL 1997 | NR | NR | 40-81 | 42-82 | 16/14 | 19/11 | NR | Gypenosides tablet (40mg) po, tid. | Red yeast rice (Zhibituo) 1.05g, po, tid. | NR | 4w | normalization of lipid levels |
| Lu YJ 2005 | Outpatient | NR | 28-76 | 30-75 | 26/24 | 25/25 | NR | Gynostemma pentaphyllum tea 30g in boiling water to drink+N-3 fatty acids~~Doxicon~~ 0.5g, po ,tid. | N-3 fatty acids~~Doxicon~~ 0.5g, po ,tid. | NR | 3m | Effective rate |
| Lu ZL 1996 | Outpatient+Inpatiet | NR | mean56.9(range37-70) | mean56.9(range37-70) | 63 | 58 | Hypertension and coronary heart disease. | Gypenosides tablet 60mg po bid | Red yeast rice (Xuezhikang capsule) 0.6g po bid | Continue to take drugs that do not affect lipid metabolism. | 8w | TC, TG, LDL-C, HDL-C, Effective rate, AE |
| Peng JW 2010 | NR | NR | 52.8(44-67) | 54.2(42-68) | 27/15 | 28/16 | Hypertension, coronary heart disease, diabetes, fat liver, and hypothyroidism | Gypenosides 40mg, po tid. | Fenofibrate 10mg, po, tid. | Control diet, increase physical activity, control blood pressure and blood glucose. | 4w | TC, TG, LDL-C, HDL-C, AE |
| Shi LW 2016 | Outpatient | Government | 52.72±9.41 | 54.94±8.67 | 24/38 | 22/41 | Hypertension, coronary heart disease, diabetes, and stroke. | Gypenosides 60mg po tid | Simvastatin capsule 20mg, po, qn | NR | 12w | TC, TG, LDL-C, HDL-C, Effective rate, AE |
| Shi M 2016 | NR | NR | 52.05±7.93 | 51.45±8.12 | 178 | | Diabetes | Gynostemma pentaphyllum powder 6g+Metformin 0.5g+Atorvastatin 20mg, po tid | Metformin 0.5g+Atorvastatin 20mg, po tid | Basic diabetes education, diabetes diet, moderate exercise and other lifestyle intervention for patients with diabetes. | 8w | TC, TG, LDL-C, HDL-C |
| Wang J 1997 | NR | University | 56.4 ± 0.83 | 56.0 ± 0.50 | 73/49 | 188/136 | NR | Gypenosides tablet (0.6g), po, bid. | Red yeast rice 0.6g, po, bid. | All medications were allowed during the trial, except those that could affect serum lipids. | 8w | normalization of lipid levels, TC, TG, LDL-C, HDL-C, Effective rate, AE |
| Wang JG 2010 | Outpatient+Inpatiet | NR | 54.9±3.21(25.7-75.4) | 43.7±3.15(22.5-76.2) | 23/10 | 30/42 | Hypertension and coronary heart disease. | Gypenosides tablet 120mg po tid | Red yeast rice (Xuezhikang capsule) 0.6g po bid | Active treatment of the primary disease (hypertensive disease and coronary heart disease). | 8w | TC, TG, LDL-C, HDL-C, Effective rate, AE |
| Xing YW 2013 | Outpatient+Inpatiet | NR | 60.0±10.2  61.0±9.9 62.0±9.1 | 63.0±8.9 | 44/46 | 15/15 | Hypertension, diabetes, stroke, and heart failure | Gypenosides table 60mg, po, tid Gypenosides table 120mg, po, tid; Gypenosides table 120mg po tid + Atorvastatin 20mg po qd | Atorvastatin 20mg po qd | NR | 1m | TC, TG, LDL-C, HDL-C, AE |
| Xu JH 2013 | Outpatient | NR | NR | NR | 50 | 46 | NR | Gypenosides tablet 20mg +Simvastatin capsule 20mg, po, tid | Simvastatin capsule 20mg, po, tid | NR | 8w | TC, TG, LDL-C, HDL-C, Effective rate |
| Yu PL 1997 | Outpatient | NR | 52.6±10.4(27-67) | 53.9±9.6(32-70) | 20/10 | 57/31 | No. | Gypenosides 60mg po bid | Red yeast rice (Xuezhikang capsule) 0.6g po bid | Low fat, low cholesterol diet, lifestyle remained relatively stable | 8w | TC, TG, LDL-C, HDL-C, Apo A1, Apo B, Effective rate, AE |
| Zhang Y 2000 | NR | NR | 56±9(40-74) | 59±6(42-73) | 19/34 | 24/19 | Hypertension, coronary heart disease, and stroke. | Gypenosides tablet 60mg po tid | Red yeast rice (Xuezhikang capsule) 0.6g po bid | NR | 4w | TC, TG, LDL-C, HDL-C, AE |
| Zhang YD 2000 | Outpatient | NR | 60-79 | 60-79 | 140 | 6 | Hypertension, coronary heart disease, diabetes, fat liver, and cerebral infarction. | Gypenosides tablet (60mg) po, tid; Gypenosides tablet (40mg) + Gypenosides gelatin pearl(1.35g) + N-3 fatty acids~~Doxicon~~ capsule 1.8g, po, tid. | N-3 fatty acids~~Doxicon~~ capsule 1.8g, po, tid. | NR | 60d | normalization of lipid levels |
| Zhao QP 2009 | Outpatient+Inpatiet | NR | 41.23±8.34(19-64) | 40.13±8.45(18-65) | 17/13 | 18/12 | Idiopathic nephrotic syndrome | Gypenosides capsule 60mg po tid | Simvastatin tablet 20mg po tid | Active treatment of the primary disease (hormones, anticoagulants, etc.), in addition, eating habits and lifestyle remain basically the same as before treatment. | 4w | TC, TG, LDL-C, HDL-C, AE |
| Zhou Y 2005 | NR | NR | 52±7.7(35-70) | 52±7.7(35-70) | 53 | 29 | NR | Gypenosides tablet 120mg po tid | Gemfibrozil capsule 0.6g po bid | NR | 3m | Effective rate, AE |

Note: T: Treatment group; C: Control group; M: male; F: female; y: years; m: months; w: weeks; d: days; NR: Not reported. po: peros; qd: once a day; tid: three times a day; bid: twice a day; mg: milligram; g: gram; TC: total cholesterol; TG: triglycerides; LDL-C: low-density lipoprotein cholesterol; HDL-C: high-density lipoprotein cholesterol; AE: adverse events; BP: blood pressure.
