# Supplementary material for: Gynostemma pentaphyllum for dyslipidemia: A systematic review of randomized controlled trials
Source: Front Pharmacol. 2022 Aug 26;13:917521. doi: 10.3389/fphar.2022.917521 (PMC9459123; doi:10.3389/fphar.2022.917521)
Supplement: Supplementary file 2 [file Table5.DOCX]

**Table 1 Search strategies of the eight databases and three clinical trials registries**

| **Databases/Registries** | **Search strategies** |
| --- | --- |
| **Pubmed** | #1 Gynostemma [mesh terms]  #2 Gynostemma* or "Gynostemma pentaphyllum*" or "pentaphyllum, Gynostemma" or gypenoside or gynoglycosides or Jiaogulan or "Jiao Gu Lan" or Jiao-Gu-Lan [All Fields]  #3 #1 OR #2  #4 Dyslipidemias [mesh terms]  #5 Triglycerides [mesh terms]  #6 Cholesterol [mesh terms]  #7 Lipoproteins [mesh terms]  #8 Apolipoprotein A-I [mesh terms]  #9 Apolipoproteins B [mesh terms]  #10 Dyslipidemia* or Cholesterol or Lipoprotein* or Lipid?emia* or Lipemia* or "Lipase D Deficiency" or Hyperlipidemia or Dysbetalipoproteinemia[All Fields]  #11 Dyslipoproteinemia* or Hyperlipid?emia* or Hyperlipemia* or Hyperprebetalipoproteinemia or Hypercholesterol?emia* or Hyperlipoprotein?emia* or Hypertriglycerid?emia* or Hyperalphalipoproteinaemia or "Broad Beta Disease" or Hypoalphalipoproteinemias or Hypobetalipoproteinemias or Hypolipoproteinemia* Or Hypoprebetalipoproteinemia[All Fields]  #12 "Total cholesterol" or TC or Triglyceride or TG or "low-density lipoprotein cholesterol" or LDL-C or "high-density lipoprotein cholesterol" or HDL-C or "Apolipoprotein A1" or "Apo A1" or "Apo A-1" or "Apolipoprotein B" or "Apo B" or "Apo B100" or "Lipoprotein a" or "Lp a"[All Fields]  #13 or/4-12  #16 #3 AND #14 |
| **Cochrane Library** | #1 Gynostemma[mesh terms]  #2 Gynostemma* or Gynostemma pentaphyllum* or pentaphyllum, Gynostemma or gypenoside or gynoglycosides or Jiaogulan or Jiao Gu Lan or Jiao-Gu-Lan[Title Abstract Keywords]  #3 #1 OR #2  #4 Dyslipidemias[mesh terms]  #5 Triglycerides[mesh terms]  #6 Cholesterol[mesh terms]  #7 Lipoproteins[mesh terms]  #8 Apolipoprotein A-I[mesh terms]  #9 Apolipoproteins B[mesh terms]  #11 Dyslipidemia* or Cholesterol or Lipoprotein* or Lipid?emia* or Lipemia* or "Lipase D Deficiency" or Hyperlipidemia or Dysbetalipoproteinemia[Title Abstract Keywords]  #12 Dyslipoproteinemia* or Hyperlipid?emia* or Hyperlipemia* or Hyperprebetalipoproteinemia or Hypercholesterol?emia* or Hyperlipoprotein?emia* or Hypertriglycerid?emia* or Hyperalphalipoproteinaemia or Broad Beta Disease or Hypoalphalipoproteinemias or Hypobetalipoproteinemias or Hypolipoproteinemia* or Hypoprebetalipoproteinemia[Title Abstract Keywords]  #13 Total cholesterol or TC or Triglyceride or TG or low-density lipoprotein cholesterol or LDL-C or high-density lipoprotein cholesterol or HDL-C or Apolipoprotein A1 or Apo A1 or Apo A-1 or Apolipoprotein B or Apo B or Apo B100 or Lipoprotein a or Lp a[Title Abstract Keywords]  #14 or/4-13  #16 #3 and #15 |
| **Embase** | #1 'Gynostemma'/exp  #2 gynostemma*  #3 'gynostemma pentaphyllum*'  #4 'pentaphyllum, gynostemma'  #5 gypenoside  #6 gynoglycosides  #7 jiaogulan  #8 'jiao gu lan'  #9 #1 OR #2 OR # OR #3 OR #4 OR #5 OR #6 OR #7 OR #8  #10 'disorders of lipid and lipoprotein metabolism'/exp  #11 'triacylglycerol'/exp  #12 'cholesterol'/exp  #13 'lipoprotein'/exp  #14 'apolipoprotein'/exp  #15 dyslipidemia*  #16 cholesterol  #17 lipoprotein*  #18 lipid?emia*  #19 lipemia*  #20 'lipase d deficiency'  #21 hyperlipidemia  #22 dysbetalipoproteinemia  #23 dyslipoproteinemia*  #24 hyperlipid?emia*  #25 hyperlipemia*  #26 hyperprebetalipoproteinemia  #27 hypercholesterol?emia  #28 hyperlipoprotein?emia*  #29 hypertriglycerid?emia  #30 hyperalphalipoproteinaemia  #31 'broad beta disease'  #32 hypoalphalipoproteinemias  #33 hypobetalipoproteinemias  #34 hypolipoproteinemia*  #35 hypoprebetalipoproteinemia  #36 'total cholesterol'  #37 tc  #38 triglyceride  #39 tg  #40 'low-density lipoprotein cholesterol'  #41 'ldl c'  #42 'high-density lipoprotein cholesterol'  #43 'hdl c'  #44 'apolipoprotein a1'  #45 'apo a1'  #46 'apo a-1'  #47 'apolipoprotein b'  #48 'apo b'  #49 'apo b100'  #50 'lipoprotein a'  #51 'lp a'  #52 OR #10-#51  #53 #9 AND #52 |
| **Web of Science** | #1 Gynostemma* or "Gynostemma pentaphyllum*" or "pentaphyllum, Gynostemma" or gypenoside or gynoglycosides or Jiaogulan or "Jiao Gu Lan" or Jiao-Gu-Lan[TS]  #2 Dyslipidemia* or Cholesterol or Lipoprotein* or Lipid?emia* or Lipemia* or Lipase D Deficiency or Hyperlipidemia or Dysbetalipoproteinemia[TS]  #3 Dyslipoproteinemia* or Hyperlipid?emia* or Hyperlipemia* or Hyperprebetalipoproteinemia or Hypercholesterol?emia* or Hyperlipoprotein?emia* or Hypertriglycerid?emia* or Hyperalphalipoproteinaemia or "Broad Beta Disease" or Hypoalphalipoproteinemias or Hypobetalipoproteinemias or Hypolipoproteinemia* Or Hypoprebetalipoproteinemia[TS]  #4 "Total cholesterol" or TC or Triglyceride or TG or "low-density lipoprotein cholesterol" or LDL-C or "high-density lipoprotein cholesterol" or HDL-C or "Apolipoprotein A1" or "Apo A1" or "Apo A-1" or "Apolipoprotein B" or "Apo B" or "Apo B100" or "Lipoprotein a" or "Lp a"[TS]  #5 or/2-4  #6 #1 and #5 |
| **China National Knowledge Infrastructure** | #1 Subject = (Jiaogulan + Qiyedan + Qiyeshen + Wuyeshen + Gongluoguodi + Yuzhaojinlong + Biandishenggen + Shenxiancao + Xiancao + Ganmancha + Ganchaman + Xiaokuyao + Changshengbulaoxiancao + Nanfangrenshen + Dierrenshen + Chaorenshen )  #2 Title Keywords Abstract = (Jiaogulan + Qiyedan + Qiyeshen + Wuyeshen + Gongluoguodi + Yuzhaojinlong + Biandishenggen + Shenxiancao + Xiancao + Ganmancha + Ganchaman + Xiaokuyao + Changshengbulaoxiancao + Nanfangrenshen + Dierrenshen + Chaorenshen )  #3 #1 OR #2  #4 Subject = (Gaoxuezhi + Gaozhixuezheng + Gaozhidanbaixuezheng + Gaodanguchunxuezheng + Gaoganyousanzhixuezheng+ Gaodimiduzhidanbai + Zhidanbaidaixiewenluan + Xuezhiyichang + Yichangxuezhi + Zhidaixieyichang +Zhidanbaiyichang + Yichangzhidanbaixuezheng + Danguchun +TG + Ganyousanzhi + TC + Gaomiduzhidanbai + HDL-C + Dimiduzhidanbai + LDL-C + Zaizhidanbai + Apo)  #5 Title Keywords Abstract = (Gaoxuezhi + Gaozhixuezheng + Gaozhidanbaixuezheng + Gaodanguchunxuezheng + Gaoganyousanzhixuezheng+ Gaodimiduzhidanbai + Zhidanbaidaixiewenluan + Xuezhiyichang + Yichangxuezhi + Zhidaixieyichang + Zhidanbaiyichang + Yichangzhidanbaixuezheng + Danguchun + TG + Ganyousanzhi + TC + Gaomiduzhidanbai + HDL-C + Dimiduzhidanbai + LDL-C + Zaizhidanbai + Apo)  #6 #4 OR #5  #7 #3 AND #6 |
| **Wanfang Database** | #1 Subject: (Jiaogulan or Qiyedan or Qiyeshen or Wuyeshen or Gongluoguodi or Yuzhaojinlong or Biandishenggen or Shenxiancao or Xiancao or Ganmancha or Ganchaman or Xiaokuyao or Changshengbulaoxiancao or Nanfangrenshen or Dierrenshen or Chaorenshen )  #2 Title or Keywords: (Jiaogulan or Qiyedan or Qiyeshen or Wuyeshen or Gongluoguodi or Yuzhaojinlong or Biandishenggen or Shenxiancao or Xiancao or Ganmancha or Ganchaman or Xiaokuyao or Changshengbulaoxiancao or Nanfangrenshen or Dierrenshen or Chaorenshen )  #3 Abstract: (Jiaogulan or Qiyedan or Qiyeshen or Wuyeshen or Gongluoguodi or Yuzhaojinlong or Biandishenggen or Shenxiancao or Xiancao or Ganmancha or Ganchaman or Xiaokuyao or Changshengbulaoxiancao or Nanfangrenshen or Dierrenshen or Chaorenshen )  #3 #1 OR #2 OR #3  #4 Subject: (Gaoxuezhi or Gaozhixuezheng or Gaozhidanbaixuezheng or Gaodanguchunxuezheng or Gaoganyousanzhixuezheng or Gaodimiduzhidanbai or Zhidanbaidaixiewenluan or Xuezhiyichang or Yichangxuezhi or Zhidaixieyichang or Zhidanbaiyichang or Yichangzhidanbaixuezheng )  #5 Title or Keywords: (Gaoxuezhi or Gaozhixuezheng or Gaozhidanbaixuezheng or Gaodanguchunxuezheng or Gaoganyousanzhixuezheng or Gaodimiduzhidanbai or Zhidanbaidaixiewenluan or Xuezhiyichang or Yichangxuezhi or Zhidaixieyichang or Zhidanbaiyichang or Yichangzhidanbaixuezheng )  #6 Abstract: (Gaoxuezhi or Gaozhixuezheng or Gaozhidanbaixuezheng or Gaodanguchunxuezheng or Gaoganyousanzhixuezheng or Gaodimiduzhidanbai or Zhidanbaidaixiewenluan or Xuezhiyichang or Yichangxuezhi or Zhidaixieyichang or Zhidanbaiyichang or Yichangzhidanbaixuezheng or Danguchun or TG or Ganyousanzhi or TC or Gaomiduzhidanbai or HDL-C or Dimiduzhidanbai or LDL-C or Zaizhidanbai or Apo)  #7 #4 OR #5 OR #6  #8 #3 AND #7 |
| **Chinese Scientific Journal Database** | #1 Title or Keywords = (Jiaogulan or Qiyedan or Qiyeshen or Wuyeshen or Gongluoguodi or Yuzhaojinlong or Biandishenggen or Shenxiancao or Xiancao or Ganmancha or Ganchaman or Xiaokuyao or Changshengbulaoxiancao or Nanfangrenshen or Dierrenshen or Chaorenshen )  #2 Abstract = (Jiaogulan or Qiyedan or Qiyeshen or Wuyeshen or Gongluoguodi or Yuzhaojinlong or Biandishenggen or Shenxiancao or Xiancao or Ganmancha or Ganchaman or Xiaokuyao or Changshengbulaoxiancao or Nanfangrenshen or Dierrenshen or Chaorenshen )  #3 #1 OR #2  #4 Title or Keywords = (Gaoxuezhi or Gaozhixuezheng or Gaozhidanbaixuezheng or Gaodanguchunxuezheng or Gaoganyousanzhixuezheng or Gaodimiduzhidanbai or Zhidanbaidaixiewenluan or Xuezhiyichang or Yichangxuezhi or Zhidaixieyichang or Zhidanbaiyichang or Yichangzhidanbaixuezheng or Danguchun or TG or Ganyousanzhi or TC or Gaomiduzhidanbai or HDL-C or Dimiduzhidanbai or LDL-C or Zaizhidanbai or Apo)  #5 Abstract = (Gaoxuezhi or Gaozhixuezheng or Gaozhidanbaixuezheng or Gaodanguchunxuezheng or Gaoganyousanzhixuezheng or Gaodimiduzhidanbai or Zhidanbaidaixiewenluan or Xuezhiyichang or Yichangxuezhi or Zhidaixieyichang or Zhidanbaiyichang or Yichangzhidanbaixuezheng or Danguchun or TG or Ganyousanzhi or TC or Gaomiduzhidanbai or HDL-C or Dimiduzhidanbai or LDL-C or Zaizhidanbai or Apo)  #6 #4 OR #5  #7 #3 AND #6 |
| **SinoMed** | #1 "Jiaogulan"[unserr weighted: extended] OR "Jiaogulanshu"[unserr weighted: extended] OR "Jiaogulanzaodai"[unserr weighted: extended]  #2 "Jiaogulan"[common field: smart] OR "Qiyedan"[common field: smart] OR "Qiyeshen"[common field: smart] OR "Wuyeshen"[common field: smart] OR "Gongluoguodi"[common field: smart] OR "Yuzhaojinlong"[common field: smart] OR "Biandishenggen"[common field: smart] OR "Shenxiancao"[common field: smart] OR "Xiancao"[common field: smart] OR "Ganmancha"[common field: smart] OR "Ganchaman"[common field: smart] OR "Xiaokuyao"[common field: smart] OR "Changshengbulaoxiancao"[common field: smart] OR "Nanfangrenshen"[common field: smart] OR "Dierrenshen"[common field: smart] OR "Chaorenshen"[common field: smart]  #3 #1 OR #2  #4 "Xuezhiyichang"[unserr weighted: extended]  #5 "Gaoxuezhi"[common field: smart] OR "Gaozhixuezheng"[common field: smart] OR "Gaozhidanbaixuezheng"[common field: smart] OR "Gaodanguchunxuezheng"[common field: smart] OR "Gaoganyousanzhixuezheng"[common field: smart] OR "Gaodimiduzhidanbai"[common field: smart] OR "Zhidanbaidaixiewenluan"[common field: smart] OR "Xuezhiyichang"[common field: smart] OR "Yichangxuezhi"[common field: smart] OR "Zhidaixieyichang"[common field: smart] OR "Zhidanbaiyichang"[common field: smart] OR "Yichangzhidanbaixuezheng"[common field: smart] OR "Danguchun"[common field: smart] OR "TG"[common field: smart] OR "Ganyousanzhi"[common field: smart] OR "TC"[common field: smart] OR "Gaomiduzhidanbai"[common field: smart] OR "HDL-C"[common field: smart] OR "Dimiduzhidanbai"[common field: smart] OR "LDL-C"[common field: smart] OR "Zaizhidanbai"[common field: smart] OR "Apo"[common field: smart]  #6 #4 OR #5  #7 #3 AND #6 |
| **The World Health Organization International Clinical Trials Registry Platform** | #1 Title: Gynostemma* OR "Gynostemma pentaphyllum*" OR "pentaphyllum, Gynostemma" OR gypenoside OR gynoglycosides OR Jiaogulan OR "Jiao Gu Lan" OR Jiao-Gu-Lan  #2 Intervention: Gynostemma* OR "Gynostemma pentaphyllum*" OR "pentaphyllum, Gynostemma" OR gypenoside OR gynoglycosides OR Jiaogulan OR "Jiao Gu Lan" OR Jiao-Gu-Lan  #3 #1 OR #2 |
| **ClinicalTrials.gov** | #1 Other terms Gynostemma* OR "Gynostemma pentaphyllum*" OR "pentaphyllum, Gynostemma" OR gypenoside OR gynoglycosides OR Jiaogulan OR "Jiao Gu Lan" OR Jiao-Gu-Lan |
| **The Chinese Clinical Trial Registry** | #1 Intervention: Jiaogulan OR Qiyedan OR Qiyeshen OR Wuyeshen OR Gongluoguodi OR Yuzhaojinlong OR Biandishenggen OR Shenxiancao OR Xiancao OR Ganmancha OR Ganchaman OR Xiaokuyao OR Changshengbulaoxiancao OR Nanfangrenshen OR Dierrenshen OR Chaorenshen |

| **Table 2 Summary of the *Gynostemma pentaphyllum* and red yeast rice** **preparations used in the included trials** | | | |
| --- | --- | --- | --- |
| **Study ID** | Drug [Species; Drug name] | Source | Preparation |
| Chen HJ 2001 | Red yeast rice (Zhibituo tablet) [Aspergillaceae; Fermentum Rubrum] | NR | NA |
|  | Gypenosides tablet [Cucurbitaceae; *Gynostemma pentaphyllum* (Thunb.) Makino] | NR | NA |
| Chen HW 1998 | Gypenosides tablet [Cucurbitaceae; *Gynostemma pentaphyllum* (Thunb.) Makino] | Guangzhou Baiyunshan Pharmaceutical CO., LTD | NA |
| Fu GX 2000 | Red yeast rice (Zhibituo tablet) [Aspergillaceae; Fermentum Rubrum] | NR | NA |
|  | Gypenosides tablet [Cucurbitaceae; *Gynostemma pentaphyllum* (Thunb.) Makino] | NR | NA |
| Huang XP 2006 | Gypenosides tablet [Cucurbitaceae; *Gynostemma pentaphyllum* (Thunb.) Makino] | Guangzhou Baiyunshan Pharmaceutical CO., LTD | NA |
| Jeenduang N 2017 | Hibiscus sabdariffa L. tea [Malvaceae; Hibiscus cannabinus L.] | NR | NA |
|  | *Gynostemma pentaphyllum* tea [Cucurbitaceae; *Gynostemma pentaphyllum* (Thunb.) Makino] | NR | NA |
| Li HC 2001 | Gypenosides capsule [Cucurbitaceae; *Gynostemma pentaphyllum* (Thunb.) Makino] | Jiangxi Shangao Pharmaceutical Co., LTD | NA |
| Lin ZD 2001 | Gypenosides tablet [Cucurbitaceae; *Gynostemma pentaphyllum* (Thunb.) Makino] | Ankang Zhengda Pharmaceutical Co. LTD | NA |
| Liu CL 1997 | Red yeast rice (Zhibituo tablet) [Aspergillaceae; Fermentum Rubrum] | Chengdu Diao Jiuhong Pharmaceutical Factory | NA |
|  | Gypenosides tablet [Cucurbitaceae; *Gynostemma pentaphyllum* (Thunb.) Makino] | NR | NA |
| Lu YJ 2005 | *Gynostemma pentaphyllum* tea [Cucurbitaceae; *Gynostemma pentaphyllum* (Thunb.) Makino] | NR | NA |
| Lu ZL 1996 | Red yeast rice (Xuezhikang capsule) [Aspergillaceae; Fermentum Rubrum] | Peking University Weixin Biological Technology Co., LTD | Xuezhikang capsule preparation^a^ |
|  | Gypenosides tablet [Cucurbitaceae; *Gynostemma pentaphyllum* (Thunb.) Makino] | Ankang Zhengda Pharmaceutical Co. LTD | NA |
| Peng JW 2010 | *Gynostemma pentaphyllum* Makino [Cucurbitaceae; *Gynostemma pentaphyllum* (Thunb.) Makino] | NR | NA |
| Shi LW 2016 | Gypenosides tablet [Cucurbitaceae; *Gynostemma pentaphyllum* (Thunb.) Makino] | Ankang Zhengda Pharmaceutical Co. LTD | NA |
| Shi M 2016 | *Gynostemma pentaphyllum* powder [Cucurbitaceae; *Gynostemma pentaphyllum* (Thunb.) Makino] | Zhejiang Ninghai Traditional Chinese Medicine Hospital | Harvest in autumn and dry in the sun. |
| Wang J 1997 | Red yeast rice [Aspergillaceae; Fermentum Rubrum] | NR | Red yeast rice preparation.^B^ |
|  | Gypenosides tablet [Cucurbitaceae; *Gynostemma pentaphyllum* (Thunb.) Makino] | NR | NA |
| Wang JG 2010 | Red yeast rice (Xuezhikang capsule) [Aspergillaceae; Fermentum Rubrum] | Peking University Weixin Biological Technology Co., LTD | Xuezhikang capsule preparation^A^ |
|  | Gypenosides tablet [Cucurbitaceae; *Gynostemma pentaphyllum* (Thunb.) Makino] | Ankang Zhengda Pharmaceutical Co. LTD | NA |
| Xing YW 2013 | Gypenosides tablet [Cucurbitaceae; *Gynostemma pentaphyllum* (Thunb.) Makino] | NR | NA |
| Xu JH 2013 | Gypenosides tablet [Cucurbitaceae; *Gynostemma pentaphyllum* (Thunb.) Makino] | Ankang Zhengda Pharmaceutical Co. LTD | NA |
| Yu PL 1997 | Red yeast rice (Xuezhikang capsule) [Aspergillaceae; Fermentum Rubrum] | NR | Xuezhikang capsule preparation^A^ |
|  | Gypenosides tablet [Cucurbitaceae; *Gynostemma pentaphyllum* (Thunb.) Makino] | NR | NA |
| Zhang Y 2000 | Red yeast rice (Xuezhikang capsule) [Aspergillaceae; Fermentum Rubrum] | Peking University Weixin Biological Technology Co., LTD | Xuezhikang capsule preparation^A^ |
|  | Gypenosides tablet [Cucurbitaceae; *Gynostemma pentaphyllum* (Thunb.) Makino] | NR | NA |
| Zhang YD 2000 | Gypenosides tablet [Cucurbitaceae; *Gynostemma pentaphyllum* (Thunb.) Makino] | Ankang Zhengda Pharmaceutical Co. LTD | NA |
| Zhao QP 2009 | Gypenosides soft capsule[Cucurbitaceae; *Gynostemma pentaphyllum* (Thunb.) Makino] | Xi 'an Datang Pharmaceutical Group Co. LTD | NA |
| Zhou Y 2005 | Gypenosides tablet [Cucurbitaceae; *Gynostemma pentaphyllum* (Thunb.) Makino] | Ankang Zhengda Pharmaceutical Co. LTD | NA |

**Table 3 Subgroup analysis of total cholesterol, triglyceride, low-density lipoprotein cholesterol, and high-density lipoprotein cholesterol**

| **Outcome** | **Experimental intervention** | **Control intervention** | **Subgroup title** | **Subgroups** | **No. of studies** | **No. of participants** | **Statistical method** | **Effect size**  **Mean difference (mmol/L)** |
| --- | --- | --- | --- | --- | --- | --- | --- | --- |
| **Total cholesterol** | *Gynostemma Pentaphyllum* | Lipid-lowering agents | Treatment duration | No more than 8 weeks | 4 | 276 | IV, Random, 95% CI | 0.69[0.09, 1.29] |
|  |  |  |  | More than 8 weeks | 3 | 215 | IV, Random, 95% CI | 0.22[-0.90, 1.33] |
|  |  |  | Basic treatment | Combined with basic treatment | 3 | 186 | IV, Random, 95% CI | 0.77[-0.18, 1.72] |
|  |  |  |  | Combined without basic treatment | 4 | 305 | IV, Random, 95% CI | 0.35[-0.39, 1.10] |
|  |  |  | Comorbidities | With comorbidities | 6 | 431 | IV, Random, 95% CI | 0.57 [-0.00, 1.15] |
|  |  |  |  | Without comorbidity | 1 | 60 | IV, Random, 95% CI | 0.13[-0.25, 0.51] |
|  | *Gynostemma Pentaphyllum* | Red yeast rice extracts | Treatment duration | No more than 8 weeks | 4 | 698 | IV, Random, 95% CI | 0.39[-0.15, 0.93] |
|  |  |  |  | More than 8 weeks | 1 | 160 | IV, Random, 95% CI | 1.66[1.33, 1.99] |
|  |  |  | Basic treatment | Combined with basic treatment | 3 | 621 | IV, Random, 95% CI | 0.41[-0.33, 1.15] |
|  |  |  |  | Combined without basic treatment | 2 | 237 | IV, Random, 95% CI | 0.98[-0.35, 2.30] |
|  |  |  | Comorbidities | With comorbidities | 4 | 412 | IV, Random, 95% CI | 0.54[-0.13, 1.21] |
|  |  |  |  | Without comorbidity | 1 | 446 | IV, Random, 95% CI | 1.01[0.95, 1.08] |
|  | *Gynostemma Pentaphyllum* plus lipid-lowering agents | Lipid-lowering agents | Treatment duration | No more than 8 weeks | 3 | 334 | IV, Random, 95% CI | -1.13[-2.59, 0.33] |
|  |  |  |  | More than 8 weeks | 1 | 30 | IV, Random, 95% CI | -0.78[-1.49, -0.07] |
|  |  |  | Basic treatment | Combined with basic treatment | 3 | 186 | IV, Random, 95% CI | -0.57[-0.79, -0.35] |
|  |  |  |  | Combined without basic treatment | 1 | 178 | IV, Random, 95% CI | -2.41[-2.57, -2.25] |
|  |  |  | Comorbidities | With comorbidities | 3 | 268 | IV, Random, 95% CI | -1.20[-2.70, 0.29] |
|  |  |  |  | Without comorbidity | 1 | 96 | IV, Random, 95% CI | -0.58[-0.84, -0.32] |
| **Triglyceride** | *Gynostemma Pentaphyllum* | Lipid-lowering agents | Treatment duration | No more than 8 weeks | 4 | 276 | IV, Random, 95% CI | 0.38[-0.00, 0.77] |
|  |  |  |  | More than 8 weeks | 3 | 215 | IV, Random, 95% CI | -0.25[-0.78, 0.28] |
|  |  |  | Basic treatment | Combined with basic treatment | 3 | 186 | IV, Random, 95% CI | 0.18[-0.25, 0.61] |
|  |  |  |  | Combined without basic treatment | 4 | 305 | IV, Random, 95% CI | 0.11[-0.40, 0.63] |
|  |  |  | Comorbidities | With comorbidities | 1 | 60 | IV, Random, 95% CI | 0.03[-0.21, 0.27] |
|  |  |  |  | Without comorbidity | 6 | 431 | IV, Random, 95% CI | 0.15[-0.26, 0.56] |
|  | *Gynostemma Pentaphyllum* | Red yeast rice extracts | Treatment duration | No more than 8 weeks | 4 | 698 | IV, Random, 95% CI | 0.36[-0.02, 0.74] |
|  |  |  |  | More than 8 weeks | 1 | 160 | IV, Random, 95% CI | 0.67[0.52, 0.82] |
|  |  |  | Basic treatment | Combined with basic treatment | 3 | 621 | IV, Random, 95% CI | 0.43[0.02, 0.85] |
|  |  |  |  | Combined without basic treatment | 2 | 237 | IV, Random, 95% CI | 0.41[-0.09, 0.92] |
|  |  |  | Comorbidities | With comorbidities | 4 | 412 | IV, Random, 95% CI | 0.35[0.01, 0.69] |
|  |  |  |  | Without comorbidity | 1 | 446 | IV, Random, 95% CI | 0.69[0.67, 0.71] |
|  | *Gynostemma Pentaphyllum* plus lipid-lowering agents | Lipid-lowering agents | Treatment duration | No more than 8 weeks | 3 | 334 | IV, Random, 95% CI | -0.57[-1.05, -0.08] |
|  |  |  |  | More than 8 weeks | 1 | 30 | IV, Random, 95% CI | -0.89[-1.16, -0.62] |
|  |  |  | Basic treatment | Combined with basic treatment | 1 | 178 | IV, Random, 95% CI | -0.50[-0.57, -0.43] |
|  |  |  |  | Combined without basic treatment | 3 | 186 | IV, Random, 95% CI | -0.70[-1.22, -0.19] |
|  |  |  | Comorbidities | With comorbidities | 3 | 268 | IV, Random, 95% CI | -0.51[-0.86, -0.15] |
|  |  |  |  | Without comorbidity | 1 | 96 | IV, Random, 95% CI | -1.10[-1.33, -0.87] |
| **Low-density lipoprotein cholesterol** | *Gynostemma Pentaphyllum* | Lipid-lowering agents | Treatment duration | No more than 8 weeks | 4 | 276 | IV, Random, 95% CI | 0.55[0.04, 1.05] |
|  |  |  |  | More than 8 weeks | 2 | 185 | IV, Random, 95% CI | 0.61[-0.04, 1.25] |
|  |  |  | Basic treatment | Combined with basic treatment | 3 | 186 | IV, Random, 95% CI | 0.13[-0.23, 0.48] |
|  |  |  |  | Combined without basic treatment | 3 | 275 | IV, Random, 95% CI | 0.81[0.33, 1.30] |
|  |  |  | Comorbidities | With comorbidities | 5 | 401 | IV, Random, 95% CI | 0.64[0.23, 1.04] |
|  |  |  |  | Without comorbidity | 1 | 60 | IV, Random, 95% CI | 0.28[0.11, 0.45] |
|  | *Gynostemma Pentaphyllum* plus lipid-lowering agents | Lipid-lowering agents | Basic treatment | Combined with basic treatment | 1 | 178 | IV, Random, 95% CI | -0.67[-0.78, -0.56] |
|  |  |  |  | Combined without basic treatment | 2 | 156 | IV, Random, 95% CI | -0.49[-1.64, 0.65] |
|  |  |  | Comorbidities | With comorbidities | 2 | 238 | IV, Random, 95% CI | -0.32[-1.07, 0.43] |
|  |  |  |  | Without comorbidity | 1 | 96 | IV, Random, 95% CI | -1.07[-1.48, -0.66] |
| **High-density lipoprotein cholesterol** | *Gynostemma Pentaphyllum* | Lipid-lowering agents | Treatment duration | No more than 8 weeks | 4 | 276 | IV, Random, 95% CI | -0.02[-0.30, 0.26] |
|  |  |  |  | More than 8 weeks | 3 | 215 | IV, Random, 95% CI | -0.85[-2.30, 0.59] |
|  |  |  | Basic treatment | Combined with basic treatment | 3 | 186 | IV, Random, 95% CI | -0.17[-0.64, 0.31] |
|  |  |  |  | Combined without basic treatment | 4 | 305 | IV, Random, 95% CI | -0.44[-1.30, 0.42] |
|  |  |  | Comorbidities | With comorbidities | 1 | 60 | IV, Random, 95% CI | -0.03[-0.15, 0.09] |
|  |  |  |  | Without comorbidity | 6 | 401 | IV, Random, 95% CI | 0.57[0.13, 1.01] |
|  | *Gynostemma Pentaphyllum* | Red yeast rice extracts | Treatment duration | No more than 8 weeks | 3 | 621 | IV, Random, 95% CI | -0.20[-0.45, 0.04] |
|  |  |  |  | More than 8 weeks | 1 | 160 | IV, Random, 95% CI | -0.42[-0.60, -0.25] |
|  |  |  | Basic treatment | Combined with basic treatment | 3 | 621 | IV, Random, 95% CI | -0.20[-0.45, 0.04] |
|  |  |  |  | Combined without basic treatment | 1 | 160 | IV, Random, 95% CI | -0.42[-0.60, -0.25] |
|  |  |  | Comorbidities | With comorbidities | 3 | 335 | IV, Random, 95% CI | -0.30[-0.59, -0.01] |
|  |  |  |  | Without comorbidity | 1 | 446 | IV, Random, 95% CI | -0.13[-0.13, -0.12] |
|  | *Gynostemma Pentaphyllum* plus lipid-lowering agents | Lipid-lowering agents | Treatment duration | No more than 8 weeks | 3 | 334 | IV, Random, 95% CI | 0.13[0.01, 0.25] |
|  |  |  |  | More than 8 weeks | 1 | 30 | IV, Random, 95% CI | 0.14[0.03, 0.25] |
|  |  |  | Basic treatment | Combined with basic treatment | 1 | 178 | IV, Random, 95% CI | 0.23[0.16, 0.30] |
|  |  |  |  | Combined without basic treatment | 3 | 186 | IV, Random, 95% CI | 0.09[0.03, 0.15] |
|  |  |  | Comorbidities | With comorbidities | 3 | 268 | IV, Random, 95% CI | 0.17[0.08, 0.25] |
|  |  |  |  | Without comorbidity | 1 | 96 | IV, Random, 95% CI | 0.04[-0.06, 0.14] |


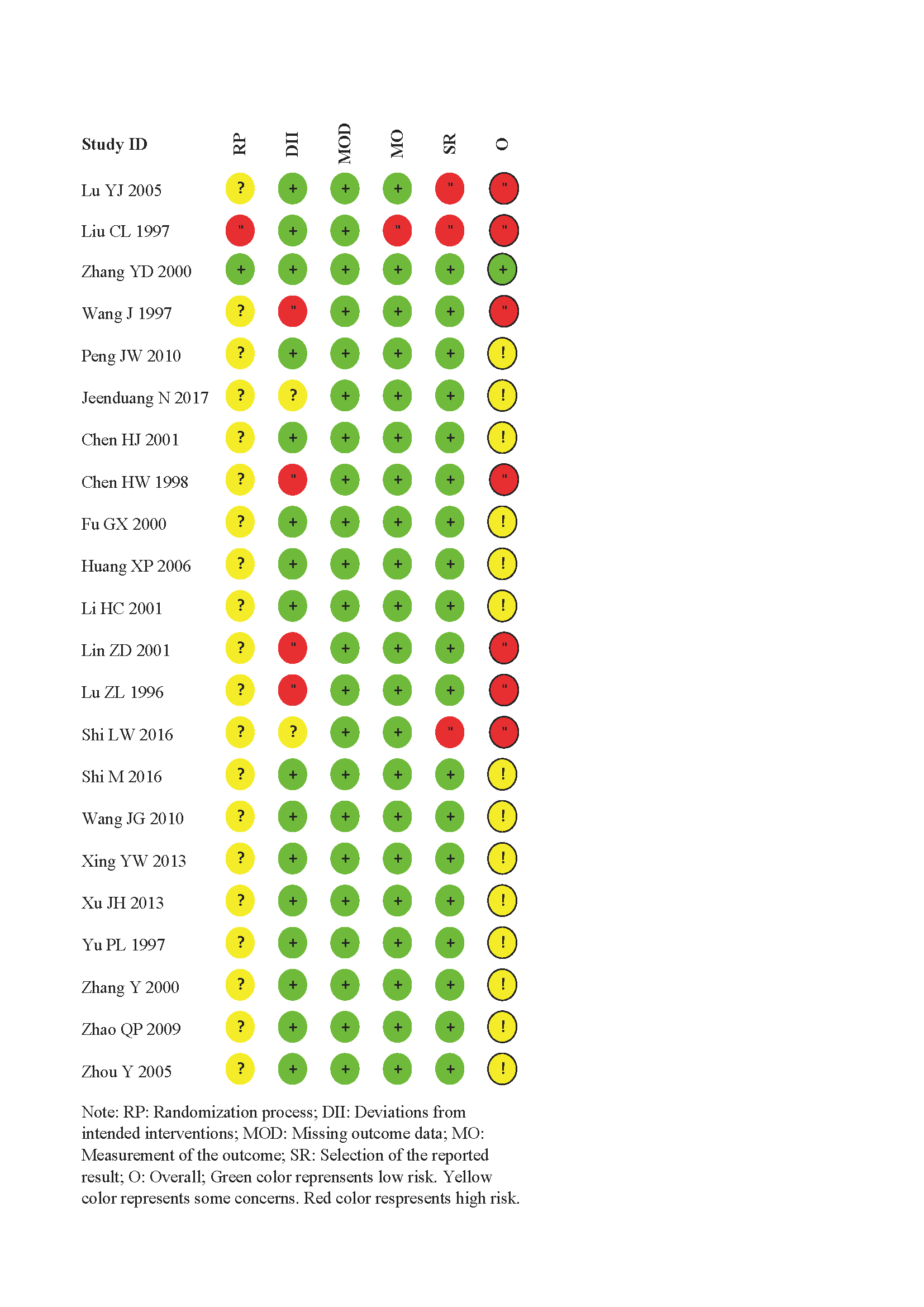


**Figure 1 Risk of bias of the included trials in details**

Note: RP: Randomization process; DII: Deviations from intended interventions; MOD: Missing outcome data; MO: Measurement of the outcome; SR: Selection of the reported result; O: Overall; Green color reprensents low risk. Yellow color represents some concerns. Red color respresents high risk.
