# Supplementary material for: Gynostemma pentaphyllum for dyslipidemia: A systematic review of randomized controlled trials
Source: Front Pharmacol. 2022 Aug 26;13:917521. doi: 10.3389/fphar.2022.917521 (PMC9459123; doi:10.3389/fphar.2022.917521)
Supplement: Supplementary file 3 [file Table3.DOCX]

**Table 2 Risk of bias of the 22 included randomized trials on** ***Gynostemea pentaphyllum* for dyslipidemia**

| **Domains** | **low risk of bias**  **n (%)** | **some concerns**  **n (%)** | **high risk of bias**  **n (%)** |
| --- | --- | --- | --- |
| **Bias arising from the randomization process** | 1 (4.5) | 20 (91) | 1 (4.5) |
| **Bias due to deviations from the intended interventions** | 16 (72.7) | 2 (9.1) | 4 (18.2) |
| **Bias due to missing outcome data** | 22 (100) | 0 (0) | 0 (0) |
| **Bias in the measurement of the outcome** | 21 (95.5) | 0 (0) | 1 (4.5) |
| **Bias in the selection of the reported result** | 19 (86.4) | 0 (0) | 3 (13.6) |
| **Overall risk of bias** | 1 (4.5) | 14 (63.6) | 7 (31.8) |
