# Supplementary material for: Gynostemma pentaphyllum for dyslipidemia: A systematic review of randomized controlled trials
Source: Front Pharmacol. 2022 Aug 26;13:917521. doi: 10.3389/fphar.2022.917521 (PMC9459123; doi:10.3389/fphar.2022.917521)
Supplement: Supplementary file 4 [file Table4.DOCX]

**Table 3 The effects of *Gynostemma Pentaphyllum* on dyslipidemia from 22 randomized controlled trials**

| **Outcome** | **Control** | **No. of studies** | **No. of participants** | **I^2^, Model (REM/FEM)** | **Effect size**  **MD mmol/L [**95% CI] | **Quality of evidence (GRADE)** |
| --- | --- | --- | --- | --- | --- | --- |
| ***Gynostemma Pentaphyllum* versus control** | | | | | | |
| **TC level** | Lipid-lowering agents | 7 | 491 | 90%, REM | 0.52[-0.01, 1.04] | Very low certainty. |
|  | Red yeast rice | 5 | 859 | 96%, REM | 0.64[0.15, 1.13] | Low certainty. |
|  | *Hibiscus sabdariffa* | 1 | 48 | 0% , FEM | -0.42[-0.97, 0.13] | Low certainty. |
| **TG level** | Lipid-lowering agents | 7 | 491 | 91%, REM | 0.13[-0.21,0.47] | Very low certainty. |
|  | Red yeast rice | 5 | 859 | 96%, REM | 0.43[0.15, 0.17] | Low certainty. |
|  | *Hibiscus sabdariffa* | 1 | 48 | 0%, FEM | -0.24[-0.62, 0.15] | Low certainty. |
| **LDL-C level** | Lipid-lowering agents | 6 | 461 | 84%, REM | 0.57[0.20, 0.93] | Low certainty. |
|  | Red yeast rice | 3 | 621 | 99%, REM | 0.37[-0.46, 1.20] | Very low certainty. |
|  | *Hibiscus sabdariffa* | 1 | 48 | 0%, FEM | -0.32[-0.77, 0.13] | Low certainty. |
| **HDL-C level** | Lipid-lowering agents | 7 | 491 | 99%, REM | -0.34[-0.93, 0.25] | Very low certainty. |
|  | Red yeast rice | 4 | 781 | 96%, REM | -0.25[-0.47, -0.04] | Low certainty. |
|  | *Hibiscus sabdariffa* | 1 | 48 | 50%, FEM | 0.10[-0.08, 0.28] | Low certainty. |
| ***Gynostemma Pentaphyllum* plus lipid-lowering agents versus lipid-lowering agents** | | | | | | |
| **TC level** | Lipid-lowering agents | 4 | 364 | 98%, REM | -1.05[-2.28, 0.19] | Very low certainty. |
| **TG level** | Lipid-lowering agents | 4 | 364 | 92%, REM | -0.65[-1.03,-0.28] | Low certainty. |
| **LDL-C level** | Lipid-lowering agents | 3 | 334 | 85%, REM | -0.57[-1.07, -0.08] | Moderate certainty. |
| **HDL-C level** | Lipid-lowering agents | 4 | 364 | 71%, REM | 0.15[0.11, 0.20] | Low certainty. |

Note: GP: *Gynostemma Pentaphyllum;* No: number; REM: random-effects model; FEM: fixed-effects model; MD: mean difference; CI: confidence interval; TC:Total cholesterol; TG:Triglyceride; LDL-C: Low-density lipoprotein cholesterol; HDL-C: High-density lipoprotein cholesterol.
